# A novel mouse cell line model reveals the tumor intrinsic and immune characteristics of EGFR-mutant lung cancer

**DOI:** 10.7150/thno.118234

**Published:** 2026-01-01

**Authors:** Yueren Yan, Jun Shang, Chunnan Liu, Yuting Chen, Yufang Bao, Yue Zhao, Fanfan Fan, Yifu Sun, Yudi Zhou, Yuke Wu, Yongbo Wang, Han Han, Haiquan Chen, Yunjian Pan

**Affiliations:** 1 Department of Thoracic Surgery and State Key Laboratory of Genetics and Development of Complex Phenotypes, Fudan University Shanghai Cancer Center, Shanghai, 200032, China.; 2 Institute of Thoracic Oncology, Fudan University, Shanghai, 200032, China.; 3 Department of Oncology, Shanghai Medical College, Fudan University, Shanghai, 200032, China.; 4 State Key Laboratory of Experimental Hematology, National Clinical Research Center for Blood Diseases, Institute of Hematology & Blood Diseases Hospital, Tianjin Institutes of Health Science, Chinese Academy of Medical Sciences & Peking Union Medical College (CAMS&PUMC), Tianjin, 301600, China.; 5 Key Laboratory of Whole-Period Monitoring and Precise Intervention of Digestive Cancer, Minhang Hospital, Fudan University, Shanghai, China.; 6 Department of Cellular and Genetic Medicine, School of Basic Medical Sciences, Fudan University, Shanghai, China.

**Keywords:** lung adenocarcinoma, EGFR, preclinical model, tyrosine kinase inhibitor, tumor microenvironment

## Abstract

**Background:** Epidermal growth factor receptor (EGFR) mutations occur frequently in lung adenocarcinoma (LUAD). However, the lack of practical preclinical models has limited our understanding of how EGFR mutations reshape the tumor immune microenvironment.

**Methods:** EGFR^E19del/E19del^; Trp53^-/-^ (EP) cell lines were established from a human EGFR exon 19 deletion mutated genetically engineered mouse model (GEMM). These cell lines were applied to generate orthotopic tumors in both immunodeficient and immunocompetent mice. Drug sensitivity assays were performed to evaluate responses to Osimertinib. Multi-omics analyses, including transcriptomic and metabolic profiling, were conducted to compare EP tumors with KRAS-mutant models and human EGFR-mutant LUAD.

**Results:** EP cell lines formed tumors in both immunodeficient and immunocompetent mice and showed sensitivity to Osimertinib comparable to human EGFR-mutant cell lines. EP tumors further developed acquired resistance to EGFR-TKI therapy, accompanied by transcriptional reprogramming marked by enhanced epithelial-mesenchymal transition (EMT) and Wnt/β-catenin signaling. Multi-omics analysis revealed that EP tumors closely recapitulate the molecular features of human EGFR-mutant LUAD, while exhibiting distinct transcriptomic and metabolic profiles compared to KRAS-mutant models. Immune profiling demonstrated a suppressed adaptive immune response in EP tumors, including reduced infiltration of tissue-resident memory T cell and increased M2-like polarization of macrophage.

**Conclusions:** This study presents a novel preclinical model that practically represents EGFR-mutant LUAD. Unlike traditional models relying on exogenous EGFR mutations, EP cells are endogenously driven by EGFR signaling and accurately reflect the immunosuppressive microenvironment observed in patients. This model provides an efficient platform for investigating mechanisms of immune evasion and for developing innovative therapeutic strategies.

## Introduction

Lung adenocarcinoma (LUAD) is the most common subtype of non-small cell lung cancer (NSCLC) 1. EGFR mutations are prevalent, accounting for over 50% of cases in Asians 2, 3 and over 10% in Caucasians with LUAD 4, 5. EGFR mutations are key oncogenic drivers in LUAD onset and progression, making them a central focus in research and treatment. Clinical trials have demonstrated that EGFR tyrosine kinase inhibitors (TKIs) offer superior outcomes compared to chemotherapy in EGFR-mutant LUAD. First-generation TKIs (Erlotinib and Gefitinib) achieve a median progression-free survival (mPFS) of 9 to 13 months, while the third-generation TKI, Osimertinib, extends mPFS to18.9 months in advanced stage patients 6, 7. However, resistance to EGFR TKIs is inevitable, leaving post-resistance treatment options an ongoing challenge 8.

Immune checkpoint inhibitor (ICI) targeting PD-1 or its ligand PD-L1 can reinvigorate exhausted T cells^9^ and has demonstrated notable clinical benefit in patients with advanced NSCLC 10-14. Yet, the efficacy of ICIs in EGFR-mutant LUAD remains controversial 15. Multiple trials using ICIs as second-line therapies have reported limited to no survival benefit for EGFR-mutant patients 10-14. Moreover, combining ICIs with TKIs failed to produce synergistic effects and often increases toxicity. Previous studies suggest that an immunosuppressive tumor microenvironment (TME) might contribute to poor immune responses observed in EGFR-mutant LUAD 16-19. However, the precise mechanisms underlying these observations remain unclear.

Current preclinical models for studying EGFR-mutant LUAD include patient-derived cell lines, organoids, and xenograft models, yet these human-derived models cannot form tumors in immunocompetent mice, limiting insights into how EGFR mutations affect tumor-immune interactions. EGFR-mutant genetically engineered mouse models (GEMMs) can spontaneously develop LUAD upon Cre induction and possess intact immune microenvironment 20-22. However, GEMMs are costly and time-consuming to maintain, and their limited suitability for genetic editing restricts mechanistic studies. By contrast, models of Kras-mutant LUAD have proven far more available. Several GEMM-derived Kras-mutant adenocarcinoma cell lines, such as KP (Kras^G12D/-^; Trp53^-/-^) and KL (Kras^G12D/-^; Lkb1^-/-^), are available 23, 24. These cell lines can form tumors in immunocompetent mice, and their genetically defined backgrounds make them highly amenable to gene editing and large-scale CRISPR library screening 25, 26.

To address the limitations in EGFR-mutant LUAD models, we developed the EP cell line, derived from a human EGFR (hEGFR) mutant GEMM, to provide a robust and versatile model for exploring the TME in EGFR-mutant LUAD.

## Materials and Methods

### Mice studies

LSL-EGFR^E19del/E19del^; Trp53^flox/flox^ mice (LSL-hEGFR^E19del^ homozygous and Trp53^flox^ homozygous) were established through crossbreeding of mice with conditional hEGFR knock-in and those with floxed Trp53 genes. To create the LSL-EGFR^E19del^ mouse model, we amplified the hEGFR exon 19 deletion (ΔE746-A750), inserted the sequence into the transgenic mouse ROSA26 locus, and regulated its expression using a LoxP-STOP-LoxP sequence. For the Trp53^flox^ model, 2 LoxP sequences were introduced into the intronic regions flanking exons 5-7 of Trp53. Genotyping primers were listed in Table S2. 6-week-old C57BL/6J mice and BALB/c-nude mice were purchased from Nanjing GemPharmatech.

For allograft model, a suspension of 1×10⁶ KP or EP cells in 100μl of ice-cold phosphate-buffered saline (PBS) was prepared and injected subcutaneously into the lower flanks of the mice. Tumor volume was evaluated every two days according to the formula: (L ×W^2^)/2, where tumor length (L) and tumor width (W) is assessed by calipers. Allograft tumors, after reaching a size of 200-300 mm³, were randomly assigned into each group for drug treatment studies. Osimertinib (AZD9291, Cat. No.: HY-15772) was purchased from MCE Chemicals. Osimertinib (5 mpk) or vehicle control (0.9% saline) was administered via gavage consecutively every 2 days.

For orthotopic model, a suspension of 1×10⁶ KP or EP cells in 100μl of ice-cold PBS was prepared and injected into each mouse intravenously. KP cells were injected into C57BL/6J mice, while EP cells were injected into Cre-negative LSL-EGFR^E19del^ mice. Micro-computed tomography (μCT) was conducted to monitor tumorigenesis and progression in orthotopic models.

### Study cohorts

The previous study generated whole genome sequencing (WGS) data for 986 lung adenocarcinoma samples and RNA-seq data for 968 samples 27. This study analyzed EGFR and KRAS co-mutations in the EP and KP groups using WGS data from 830 invasive samples. Additionally, tumor microenvironment and gene function enrichment analyses were performed on RNA-seq data from 142 EGFR&TP53 (EGFR and TP53 co-mutated) and 13 KRAS&TP53 (KRAS and TP53 co-mutated) invasive solid samples.

The flow cytometry cohort included 184 LUAD samples, which were obtained from FUSCC from September 2020 to December 2021, as previously described 28. We performed the flow cytometry cohort to study the phenotype of T cells and macrophages (FUSCC FCM cohort). Since the number of viable immune cells obtained from each tumor varied, we prioritized staining with the T cell panel when cell numbers were limited. In such cases, staining with the myeloid panel could not be performed, resulting in a smaller sample size for M2 macrophage analysis compared with CD103⁺ T cell analysis. The baseline characteristics of FUSCC FCM cohort is included in Table S1.

### Primary cell construction of EP

LSL-EGFR^E19del/E19del^; Trp53^flox/flox^ mice at 6-8 weeks were infected intranasally with 6×10^10^ vg AAV-Cre. 8 weeks after infection, lung tumor was monitored by μCT. We then euthanized the mice and dissected the lung tumors. After dissecting the tumor tissue, place it in PBS containing antibiotics and remove any surrounding normal tissue. Transfer the tumor tissue into an EP tube and mince it into small fragments. Add 1 mL of 10% FBS advanced-DMEM/F-12 medium to the tumor fragments. Then, add an additional 4 mL of 10% FBS advanced-DMEM/F-12 medium and culture the tissue at 37 °C in a 5% CO₂ environment for five days. Once cells start to migrate from the tumor fragments, refresh the medium. After 10 days, passage the cells. During the first ten passages, observe cell morphology. If fibroblasts begin to proliferate, use 0.25% trypsin to digest briefly for 30-90 s, then wash twice with PBS to reduce fibroblast accumulation. By the tenth passage, the tumor cells should be highly purified and stably passaged. The EP1 cell line was deposited at the China Center for Type Culture Collection (CCTCC, accession No: C2023394).

### Cell culture

The KP cell line was provided by Dr. Fei Li (Fudan University). PC9, A549, KLN205, and LLC cells were obtained from Cobioer Biotechnology (Nanjing). PC9, KP, and KLN205 cells were propagated in 10% FBS RPMI-1640 medium, whereas LLC cells were propagated in 10% FBS DMEM medium. Cell culture was performed in a humidified 5% CO₂ incubator set to 37°C. Cell identity was authenticated by short tandem repeat profiling by the suppliers, and mycoplasma contamination was routinely tested using PCR-based assays with specific primers.

### Establishment of Osimertinib-resistant EP1 cells

To generate Osimertinib-resistant cell lines, parental EP1 cells were continuously exposed to stepwise increases in Osimertinib concentration, beginning at 10 nM and gradually escalating until cells could tolerate micromolar levels, following previously described procedures 29. Resistant clones were maintained in medium containing 2 µM Osimertinib and were designated EP1-res.

### Immunofluorescence

Cells were plated onto sterile coverslips, washed with ice-cold PBS, and subsequently fixed with 4% paraformaldehyde for 1 h. Permeabilization was then carried out using 0.5% Triton X-100 for 15 min. Blocking was performed with 3% BSA for 1 h at 37 °C, prior to an overnight incubation with primary antibodies at 4 °C. Finally, the cells were treated with fluorophore-conjugated secondary antibodies and counterstained with DAPI. All images were captured with an Olympus confocal fluorescence microscope. Primary antibodies included KRT7 (1:1000; Abcam, Cat. No.: ab181598), KRT5 (1:1000; Abcam, Cat. No.: ab52635), EPCAM (1:1000; Abcam, Cat. No.: ab71916), EGFR (1:1000; Proteintech, Cat. No.: 18986-1-AP), CD103 (1:1000; Abcam, Cat. No.: ab224202), CD4 (1:400; Abcam, Cat. No.: ab133616), CD8 (1:1000; Abcam, Cat. No.: ab217344), CD3 (1:200; Abcam, Cat. No.: ab16669), CD206 (1:2000; Abcam, Cat. No.: ab64693), and F4/80 (1:200; CST, Cat. No.: 70076).

### Immunohistochemistry staining

Formalin-fixed, paraffin-embedded sections were processed by deparaffinization in xylene, rehydration through a graded ethanol series, and immersion in water. Antigen retrieval was performed in citrate buffer (10 mM, pH 6.0) at sub-boiling temperature for 15 min. Sections were permeabilized with 0.5% Triton X-100 for 20 min and incubated with 3% H_2_O_2_ for 10 min to quench endogenous peroxidase activity. After blocking with 3% BSA for 30 min, sections were incubated overnight with primary antibodies at 4 °C. Slides were washed with PBS, treated with secondary antibodies for 30 min, and visualized using a DAB substrate kit. Hematoxylin counterstaining was performed before dehydration and mounting. Images were captured using an Olympus DP72 microscope. Antibodies included ECAD (1:1000; Proteintech, Cat. No.: 20874-1-ap), NCAD (1:2000; Proteintech, Cat. No.: 22018-1-ap), Ki-67 (1:2000; Abcam, Cat. No.: ab15580) and TTF-1 (1:4000; HUABIO, Cat. No.: HA720067).

### Quantitative PCR

Total RNA was isolated via TRIzol reagent (Vazyme) followed by the supplier's guidelines. cDNA synthesis was performed with 1 µg RNA using the Goldenstar RT6 cDNA Synthesis Kit (TsingKe Biological Technology), which includes a DNA removal step. Quantitative PCR (qPCR) was conducted with AceQ SYBR Green Master Mix (Vazyme) on an ABI Step One Plus system (Applied Biosystems). Reaction specificity was confirmed by melting curve analysis and agarose gel electrophoresis. All reactions were performed in triplicate, and expression levels were determined by the 2⁻ΔΔCt method. All primer sequences used for qPCR are available in in Table S2.

### In vitro treatment assay

Osimertinib, XAV-939, MK-2206, Gefitinib and Afatinib were obtained from MCE. Tumor cells (3,000 cells/well) were seeded in 96-well plates in 100 µL medium. After one-day incubation, cells were treated with indicated compounds at specified concentrations. Following 72 h of drug exposure, 10% (v/v) CCK-8 reagent (Meilunbio) was added and incubated for 60 min. Absorbance at 450 nm was measured using a BioTek microplate reader. The viability of treated cells was calculated relative to control wells exposed to vehicle only.

### Western blot

Western blotting was conducted as described in earlier studies 30. Tumor cell lysates were prepared using RIPA buffer (Meilunbio) supplemented with protease and phosphatase inhibitors (MCE). Equal protein aliquots (30 μg) were subjected to electrophoresis on SDS-PAGE gels and then electroblotted onto PVDF membranes (Millipore). Processed membranes were then incubated with primary antibodies. Protein bands were detected using standard chemiluminescence methods.

The following primary antibodies were used: EGFR (1:2000; CST, Cat. No.: 4267), pEGFR (1:2000; CST, Cat. No.: 2234), ERK1/2 (1:2000; CST, Cat. No.: 4695), pERK1/2 (1:2000; CST, Cat. No.: 9101), PD-L1 (1:2000; Abclonal, Cat. No.: A20481), β-Actin (1:5000; Proteintech, Cat. No.: 66009-1-Ig), and GAPDH (1:5000; Abclonal, Cat. No.: A19056).

### Multiplex flow cytometry

Multiplex Flow Cytometry was conducted following previously established protocols 31, 32. After euthanasia, cardiac perfusion with PBS was performed. Lungs were minced and enzymatically digested at 37°C for 30 min. The mixture was then filtered through a 70 µm strainer, and erythrocytes were removed with RBC lysis buffer (BioLegend). Cell suspension was labeled with LIVE/DEAD™ Fixable Dead Cell Stain (Thermo Fisher), followed by fluorochrome-conjugated antibodies (listed in Table S3). Data were collected on a BD LSRFortessa and processed with FlowJo. Gating strategies are shown in Figure S7.

### RNA-seq analysis

Transcriptome libraries were constructed using the VAHTS Universal V6 RNA-seq Library Prep Kit followed by manufacturer's protocol. Library sequencing was conducted by OE Biotech Co., Ltd. (Shanghai). Raw FASTQ files from RNA-seq were evaluated for base quality using FastQC (v0.11.9). The raw data was aligned to the mouse genome (GRCm38.p6) and gene expression was quantified using the Hisat2-StringTie 33 pipeline. Gene annotations were obtained from GENECODE's gencode.vM23, and final expression levels were quantified using FPKM (Fragments per kilobase of exon model per million mapped fragments). Differential expression analysis was conducted using the Limma 34 package (v3.50.0), identifying differentially expressed genes (DEGs) with |Log2FC| ≥ 1 and *P* < 0.05 as significantly between two groups.

### Tumor microenvironment and gene function enrichment analysis

Tumor microenvironment cell scoring based on transcriptome data was conducted using the CIBERSORTx 35 online analysis platform. Gene expression matrices were uploaded to generate individual cell scoring matrices for each sample, and all subsequent statistical analyses were performed using these matrices. Single-sample gene enrichment scores were analyzed with the R package GSVA 36 (v1.42.0), while GSEA analyses were carried out using the R package GSEA 37 (v1.2). Gene function datasets were obtained from the GSEA database, including human markers (h.all.v7.5), canonical pathways (c2.cp.kegg.v2023.1), and murine markers (mh.all.v2024.1.Mm). The cell marker genes (Brd2, Itgae, Cd69, FosB, Nfkbid) of lung tissue-resident memory T (T_RM_) cells were extracted according to CellMarker 2.0 database. The GSVA was applied to calculate the T_RM_ cell gene set enrichment scores. The homologene R package was used to convert the Mus musculus (NCBI:txid10090) gene names in the FPKM data to their Homo sapiens (NCBI:txid9606) gene name equivalents.

### Non-targeted metabolomic data generation and analysis

Tumor samples were homogenized in liquid nitrogen and subjected to LC-MS analysis. Metabolite profiling, conducted by OE Biotech Co., Ltd. (Shanghai), was performed using an ultra-performance liquid chromatography system coupled with a high-resolution quadrupole time-of-flight mass spectrometer (UPLC-QTOF, Waters) under both positive and negative ion modes. Separation was achieved on a C18 reversed-phase column with a gradient of water and acetonitrile (both containing 0.1% formic acid).

Raw data were processed with Progenesis QI software. Peaks were aligned and normalized, and metabolites were annotated by matching RT-m/z features against HMDB, LipidMaps, and in-house reference libraries. Quality control samples were analyzed throughout the run to monitor instrument stability.

For statistical analysis, features absent in more than half of the samples were removed. Principal component analysis (PCA) was applied for data visualization and identification of discriminant metabolites. Compounds with VIP > 1.0 and p < 0.05 (Student's t-test) were considered significantly altered. Pathway enrichment analysis was performed using KEGG and Reactome databases.

### Statistics and plots

Statistical analyses for all experiments were conducted using GraphPad Prism 9 (GraphPad software). Two-tailed Student's t-tests were applied for pairwise comparisons, while one-way or two-way ANOVA followed by post hoc multiple comparisons tests were utilized for experiments involving more than two conditions.

All RNA-seq and metabolomics data analyses, as well as statistical tests, were conducted using R (v4.1.2). Intergroup differences in ssGSEA scores were assessed with the Wilcoxon test. Heatmaps were created with the R package ComplexHeatmap (v2.15.4), while boxplots and barplots were created by the ggplot2 package (v3.4.0).

### Availability of data

RNA-seq raw data have been deposited in the National Omics Data Encyclopedia (NODE) (Accession number: OEP00006358). The gene expression and metabolite expression matrices can be accessed from the Zenodo platform (https://zenodo.org/records/17059562). Genomic and transcriptomic data for TCGA-LUAD were retrieved from the GDC database (https://gdc.cancer.gov/).

## Results

### Generation of GEMM-derived hEGFR mutant lung adenocarcinoma cell lines

**Figure 1:**
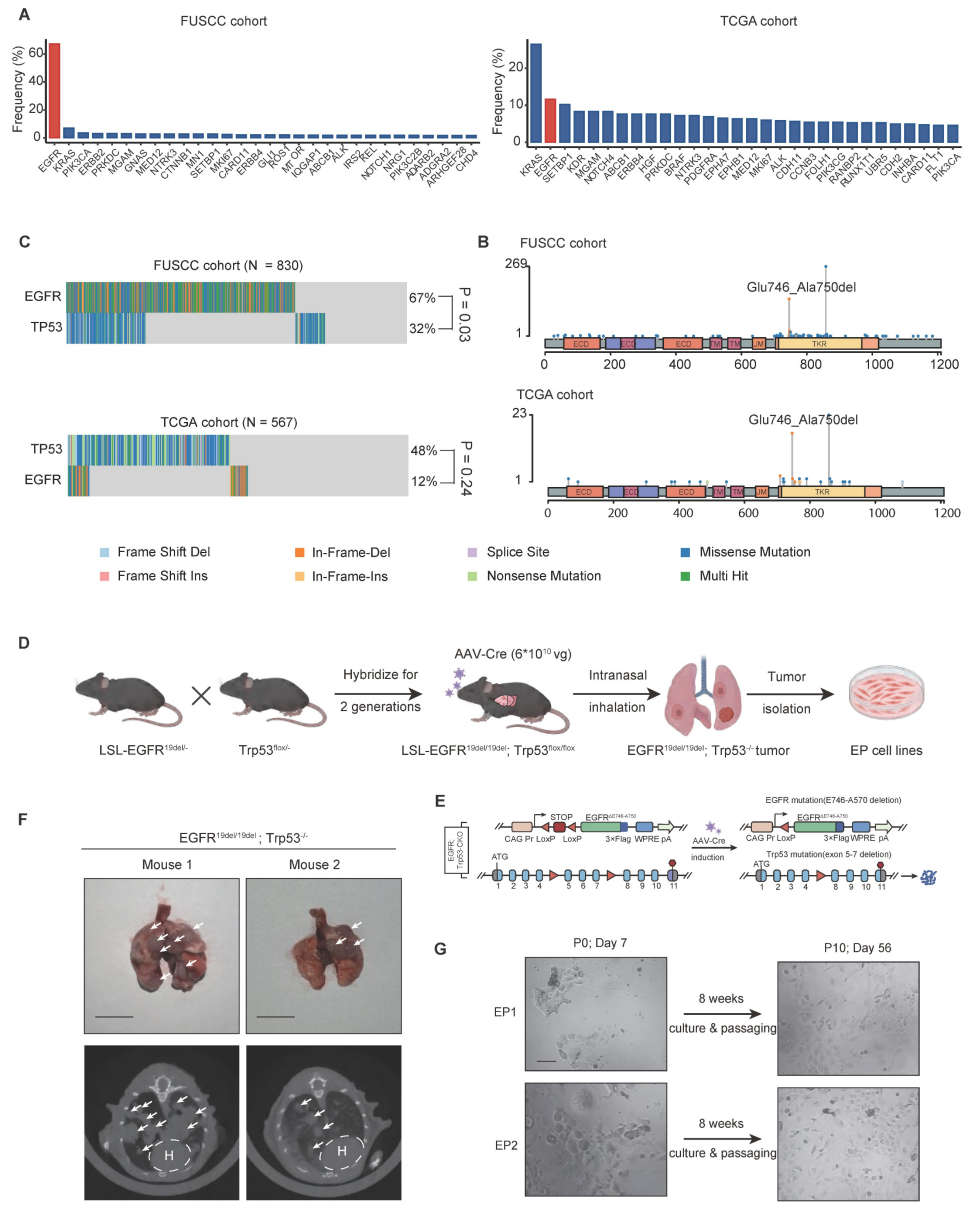
** Generation of EP cell line, a GEMM-derived hEGFR mutant LUAD cell line.** (**A**) Bar graphs showing the frequently mutated oncogenes in LUAD according to the Fudan University Shanghai Cancer (FUSCC) and TCGA cohort. EGFR is mutated in 558 of 830 LUAD samples in FUSCC cohort and in 66 of 565 LUAD samples in TCGA cohort. (**B**) Lollipop graph showing mutation profiles (truncating, missense, in-frame, and splice) of EGFR gene in the FUSCC cohort and TCGA PanCancer Atlas dataset. The mutation of ΔE746-A750 was highlighted. (**C**) Oncoplots showing EGFR and TP53 mutation profiles in LUAD patient cohorts from FUSCC and TCGA. (**D**) Schematic illustration of the construction process for the EP cell line, derived from genetically engineered mouse models (GEMMs) of hEGFR-mutant LUAD. (**E**) Illustration of conditional knock-in of the hEGFR exon 19 deletion mutation and Trp53 knock-out via Cre recombinase activation. (**F**) Morphology and computed tomography (CT) images of EGFR^E19del/E19del^; Trp53^-/-^ mice after 8 weeks of AAV-Cre inhalation, showing tumor development. Scale bar = 1 cm. (**G**) Representative brightfield images of primary EP1 and EP2 cells in culture at days 7 and 56, respectively. Scale bar = 50 μm.

EGFR is the predominantly mutated oncogenes in LUAD, ranking first (67%) in FUSCC cohort (eastern Asian) and second (12%) in TCGA cohort (Western population), according to the OncoKB Cancer Gene List 38 (Figure 1A). The main components of the EGFR protein include the extracellular domain (ECD), the transmembrane domain (TM), as well as the intracellular juxtamembrane (JM) and tyrosine kinase regions (TK). The exon 19 deletion mutation (ΔE746-A750), one of the most prevalent EGFR mutation types in LUAD, occurs in the TK domain. Like other oncogenic EGFR mutations, it leads to constitutive kinase activation and promotes tumorigenesis (Figure 1B). EGFR and TP53 co-mutations are also frequently observed in LUAD, particularly in East Asian populations. In our FUSCC cohort, 23.3% of cases harbored both mutations, compared to 6.35% in the TCGA LUAD cohort (Figure 1C). Based on this, we generated a GEMM model combining a conditional knock-in hEGFR exon 19 deletion mutation (LSL-EGFR^E19del/E19del^) with a conditional knockout of Trp53 (Trp53^flox/flox^) (Figure 1D). By crossing these two mice line for two generations, we established a double transgenic model (LSL-EGFR^E19del/E19del^; Trp53^flox/flox^), in which Cre recombinase induces the expression of hEGFR exon 19 deletion (ΔE746-A750) and deletion of Trp53 (Figure 1E). Eight weeks after intranasal inhalation of AAV-Cre in EP mice, lung tumors developed, as confirmed by CT scans and gross pathology (Figure 1F). Primary culture was performed on orthotopic lung tumors derived from two independent EP mice, result in two stable EP cell lines, EP1 and EP2 cell lines. Both EP cell lines could be long-term passaged and exhibited typical epithelial morphology (Figure 1G).

### Comprehensive and robust benchmark of EP cell lines

**Figure 2:**
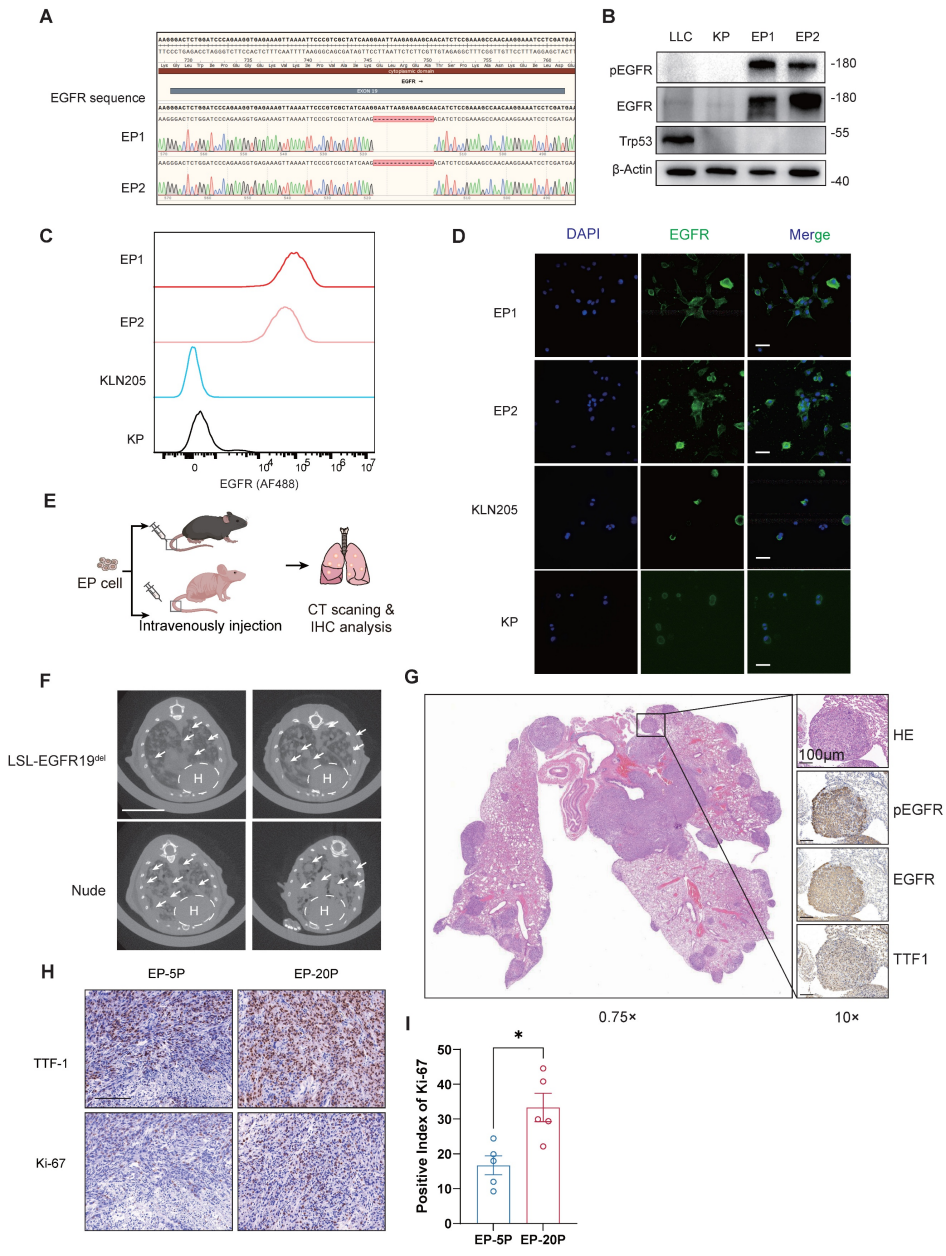
** Characterization and tumorigenic capacity of EP cell lines.** (**A**) Sanger sequencing confirming human EGFR exon 19 deletion in EP1 and EP2 cell lines. (**B**) Western blot assay showing phosphorylated and total EGFR and Trp53 protein levels in LLC, KP, EP1, and EP2 cells. Loading control: β-Actin. (**C**) Flow cytometry analysis of EGFR surface expression in EP1, EP2, KP, and KLN205 cells. (**D**) Immunofluorescence staining of EGFR (green) in EP1, EP2, KP, and KLN205 cells. Scale bar = 25 μm (**E**) Schematic illustration of EP cell intravenous injection into both immunodeficient and immunocompetent mice to assess tumorigenic potential. (**F**) Representative CT images of immunodeficient and immunocompetent mice 8 weeks post-injection with EP cells, illustrating lung tumor formation. Scale bar = 1 cm. (**G**) Histopathological images of serial lung sections from an immunocompetent mouse injected with EP cells, showing H&E and IHC staining for phosphorylated EGFR (pEGFR), EGFR, and TTF-1. Scale bar = 100 μm. (**H**) Histopathological images of subcutaneous tumors injected with early-passage (5^th^ passage, EP-5P) or late-passage (20^th^ passage, EP-20P) EP cells, showing IHC for TTF-1 and Ki-67. Scale bar = 100 μm. (**I**) Bar graphs comparing the positive index of Ki-67 between EP-5P and EP-20P. All data are mean ± SEM. *, P < 0.05. Student's t-test in (**I**).

To validate genetically, we firstly performed sanger sequencing to verify the mutant hEGFR cDNA sequences (Figure 2A). The successful genomic knockout of Trp53 was confirmed by PCR analysis at exons 2-3 for assessing the cutting efficiency of Cre Recombinase (Figure S1A). We then performed Western Blot assay to evaluate the expression levels of EGFR, phosphorylated EGFR, and Trp53 proteins across EP1, EP2, KP, and LLC cell lines, demonstrating EGFR activation and Trp53 loss of function in EP cells (Figure 2B). Immunofluorescence (IF) and Flow Cytometry (FC) analysis consistently showed significantly elevated cell surface EGFR expression in EP cell lines (Figure 2C and D).

To verify the histological origin of EP cell lines as lung adenocarcinoma, we evaluated the expression of adenocarcinoma markers Ttf1 and Napsa, as well as squamous cell carcinoma markers Krt5 and Np60 by qPCR (Figure S1B). Compared to the mouse squamous cell carcinoma cell line KLN-205, EP cells exhibited markedly higher expression of Ttf1 and Napsa, while the expression of Krt5 and Np60 was significantly lower. We then evaluated the expression of KRT5 and KRT7 at protein level via IF and FC. Consistently, EP1 and EP2 show high expression of KRT7 and low expression of KRT5 at protein level, further supporting the adenocarcinoma origin (Figure S1C and D).

### EP cell lines form orthotopic lung tumors in both immunodeficient and immunocompetent mouse models

The EP cell lines were originated from the C57BL/6J strain, making them genetically compatible with immunocompetent background. To investigate the capability of tumor formation, we injected EP1 cell line via the tail vein into both nude mice and LSL-EGFR^E19del^ mice (Figure 2E). After 10 weeks, we detected the presence of lung tumors in both groups using CT scans (Figure 2F). IHC analysis of lung tissues from immunocompetent mice confirmed the activation of EGFR signaling and LUAD histology, as evident by strong positive staining of pEGFR, EGFR and TTF1 respectively (Figure 2G). We injected early-passage (5^th^ passage, EP-5P) and late-passage (20^th^ passage, EP-20P) EP cells subcutaneously into immunocompetent mice and performed histological analysis. Both EP-5P and EP-20P tumors expressed TTF-1 and maintained LUAD morphology (Figure 2H). Notably, Ki-67 staining showed a higher proliferative index in EP-20p tumors compared to EP-5p tumors (Figure 2I), which may reflect the outgrowth of more proliferative clones during serial passaging. These results demonstrate that EP cell lines are capable of forming orthotopic lung tumors in both immunodeficient and immunocompetent mice.

Furthermore, to directly assess the impact of host immunity, we established orthotopic lung adenocarcinoma models via tail vein injection in immunocompetent (n = 6) and immunodeficient (n = 6) mice (Figure S2A). Survival analysis revealed that immunocompetent mice survived significantly longer than the nude group (Figure S2B), while there were no metastases detected in the liver or brain of either group (Figure S2C). Consistently, in subcutaneous tumor models, tumors in immunocompetent mice grew at a markedly slower rate compared with those in immunodeficient mice (Figure S2D-F). These findings emphasize the essential role of immune surveillance in suppressing EP tumor growth in vivo. Based on microenvironmental characteristics of subcutaneous tumor models, tail vein models in immunocompetent mice, and GEMM spontaneous tumor models, CD8^+^ T cells exhibited higher activity in tail vein injection and GEMM compared to immunocompetent subcutaneous tumor mice, while M2 macrophages associated with immunosuppression showed the opposite trend (Figure S2G). These results further explain the tumorigenic characteristics of the EP1 model.

### EP is sensitive to Osimertinib both in vitro and in vivo

**Figure 3:**
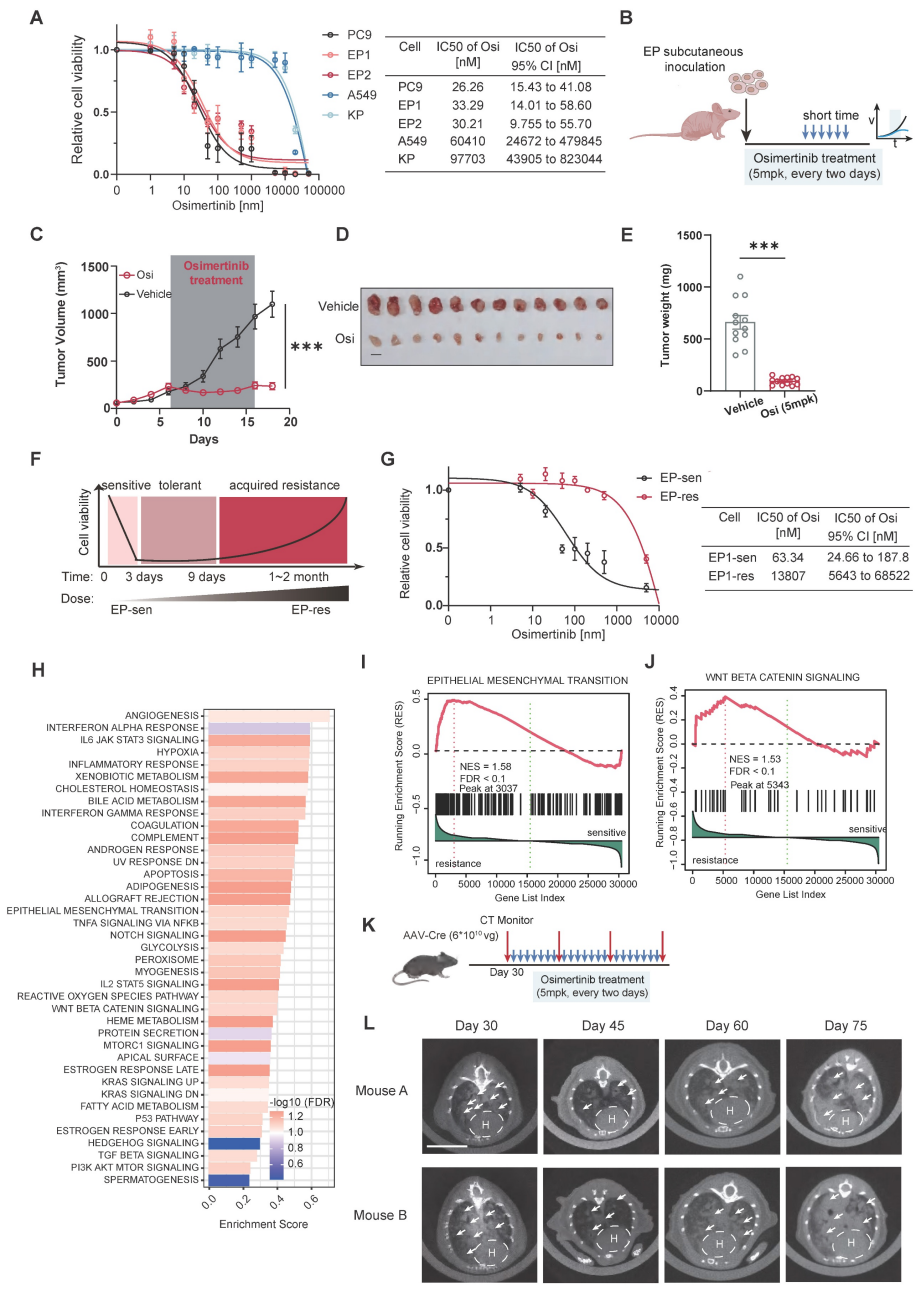
** EP cell lines simulate the response of EGFR-TKI in EGFR-mutant LUAD both in vitro and in vivo.** (**A**) Dose response curve of PC9, EP1, EP2, A549 and KP cells treated with Osimertinib. The IC50 value with 95% confidence interval of Osimertinib was presented in the right of the panel. n = 2 biological replicates. (**B**) Schematic illustration of allograft formation and treatment. Nude mice transplanted subcutaneously with EP cells were treated with vehicle (n =12) or Osimertinib (n = 12) (Osi, 5 mpk every two days) for 10 days. (**C-E**) Growth curve (**C**), end point illustration (**D**) and tumor weight (**E**) of EP1 allograft tumors. Scale bar = 1cm. (**F**) Schematic illustration of continuous exposure to stepwise increases in Osimertinib concentration to generate EP (EP-sen) cell lines with acquired resistance to Osimertinib (EP-res). (**G**) Dose response curve of EP-sen, EP-res cells treated with Osimertinib. The IC50 value with 95% confidence interval of Osimertinib was presented in the right of the panel. n =2 biological replicates. (**H**) The bar plot shows hallmark enrichment in the resistant group, with the horizontal axis representing enrichment scores and colors indicating FDR values. (**I-J**) Resistance-associated hallmarks, including EMT (**I**) and WNT BETA CATENIN SIGNALING (**J**), are emphasized in the GSEA enrichment analysis results. (**K-L**) Schematic illustration (**K**) and representative CT images (**L**) of 2 EGFR^E19del/E19del^; Trp53^-/-^ GEMM mice treated with prolonged Osimertinib administration. LSL- EGFR^E19del/E19del^; Trp53^flox/flox^ mice were infected intranasally with 6×10^10^ vg AAV-Cre, and were subsequently treated with vehicle or Osimertinib (Osi, 5 mpk every two days) at Day 30. Lung tumors were monitored by μCT scanning every two weeks. Scale bar = 1cm. All data are mean ± SEM. *, P < 0.05; **, P < 0.01; ***, P < 0.001. Two-way ANOVA with Tukey's test in (**C**), Student's t-test in (**E**).

The hEGFR exon 19 deletion mutation carried in EP cells is a known sensitizing mutation for EGFR-TKI therapy in LUAD. Hence, we examined the inhibitory efficacy of Osimertinib on EP1, EP2, PC9 (harboring *EGFR*^△E746-A750^), as well as KRAS-mutant cell lines KP and A549 (harboring KRAS^G12D^). Two EP cell lines (EP1 and EP2) exhibited sensitivity to Osimertinib comparable to PC9, with relatively low IC_50_ value. However, KP and A549 cells showed significantly higher IC₅₀ values, indicating their resistance to Osimertinib (Figure 3A). In addition, we evaluated the response of EP cells to Gefitinib and Afatinib. Similar to PC9 cells, EP cells demonstrated marked sensitivity to both first- and second-generation TKIs, whereas A549 cells exhibited limited responsiveness (Figure 3A and B). We subsequently performed Western Blot to assess the effects of Osimertinib treatment on phosphorylated EGFR and downstream phosphorylated ERK at different time points in the EP1 and PC9 cell lines. The dynamic variations of phosphorylated EGFR and ERK were similar in EP1 and PC9 (Figure S3C). For in vivo validation of EGFR-TKI, we subcutaneously implanted EP cells into the lower flanks of nude mice. Mice were randomized to receive either vehicle or Osimertinib (5mpk, every two days) (Figure 3B). Notably, Osimertinib significantly suppressed tumor growth relative to vehicle (Figure 3C-E). Collectively, these results demonstrated that the EP cell line is sensitive to Osimertinib, effectively recapitulation the EGFR-TKI response observed in human EGFR-mutant LUAD and further validating its utility as a practical model for EGFR-TKI therapy investigation.

### Leveraging the EP model to simulate Osimertinib resistance in EGFR-Mutant LUAD

LUAD Patients harboring EGFR-sensitive mutations inevitably develop resistance to TKI therapy after long-term treatment 8. To investigate whether the EP model can replicate the process of acquired resistance to Osimertinib, we exposed parental EP cells to gradually increasing doses of Osimertinib in vitro (Figure 3F). After 30 days, we observed that EP cells developed resistance to Osimertinib (EP-res) and were able to grow stably in medium containing 2 μM Osimertinib. These resistant cells (EP-res) exhibited a significantly higher IC₅₀ compared to the drug-sensitive parental cells (EP-sen), confirming the development of acquired resistance (Figure 3G). We next performed transcriptomic analysis to compare the molecular differences between EP-res (n = 3) and EP-sen (n = 3). 476 genes were significantly up-regulated in EP-res group, while 222 genes were significantly down-regulated in EP-Sen group, indicating significant transcriptional reprogramming (Figure S3D-F). To investigate the functional mechanisms of TKI resistance, we conducted functional enrichment analyses comparing the Res and Sen groups. The results indicated that 37 hallmarks might be associated with TKI resistance in EP cell lines (Figure 3H, FDR < 0.25). To note, the epithelial-mesenchymal transition (EMT), Wnt-β-catenin, and PI3K-AKT-mTOR signaling pathways were significantly enriched in the EP-res group (Figure 3I and J), consistent with known mechanisms of EGFR-TKI resistance 8, 39-41. To functionally validate these findings, we treated EP-res cells with the AKT inhibitor, MK-2206, and the WNT inhibitor, XAV-939, at various concentrations. MK-2206 alone could inhibit EP-res cell proliferation at low doses and, importantly, re-sensitized the cells to Osimertinib, showing a synergistic inhibitory effect when used in combination (Figure S3G). XAV-939 alone had limited impact on the growth of EP-res cell, however, its combination with Osimertinib partially reversed the resistance phenotype (Figure S3H).

To further assess whether the EP model can also develop TKI resistance in vivo, we extended the treatment duration of Osimertinib in the EGFR^E19del/E1del^; Trp53^-/-^ GEMM model (Figure 3K and L) and EP subcutaneous tumor model (Figure S4A). Remarkably, following an initial growth inhibition by Osimertinib, the treated EP tumors entered a plateau phase and then subsequently resumed growth (Figure S4B), indicating the EGFR-TKI tolerance in the EP tumors. By comparing gene expression changes in subcutaneous tumors of mice before and after drug resistance, the Wnt pathway was also found to be activated in the TKI-resistant group (Figure S4C and 4D). Also, IHC analysis further validated that Osimertinib resistant tumors were highly expressed EMT marker, N-cadherin (Figure S4E).

Since the enrichment of inflammation-related pathways in Osimertinib-resistant (EP-res) cells suggested a potential shift in the tumor immune microenvironment (TIME) (Figure 3H), raising the intriguing possibility that Osimertinib resistance may render tumors more susceptible to ICI. We conducted animal studies using immunocompetent mice bearing subcutaneous EP-res tumors. We treated mice with Osimertinib, anti-PD-1 antibody (α-PD1), or their combination. Compared with the vehicle group, none of the treatments—either as monotherapy or in combination—achieved significant tumor inhibition (Figure S5A-C). Moreover, IF staining demonstrated that Osimertinib, α-PD1, or their combination had limited effect on infiltration of CD4⁺ or CD8⁺ T cells relative to vehicle group (Figure S5D-F).

Together, these findings demonstrate that the EP model accurately recapitulates both in vitro and in vivo resistance to Osimertinib, offering an efficient and practical model to investigate resistance mechanisms and explore new therapeutic strategies in EGFR-mutant LUAD.

### EP models exhibit distinct oncogenic signaling and microenvironment of EGFR-mutant LUAD

**Figure 4:**
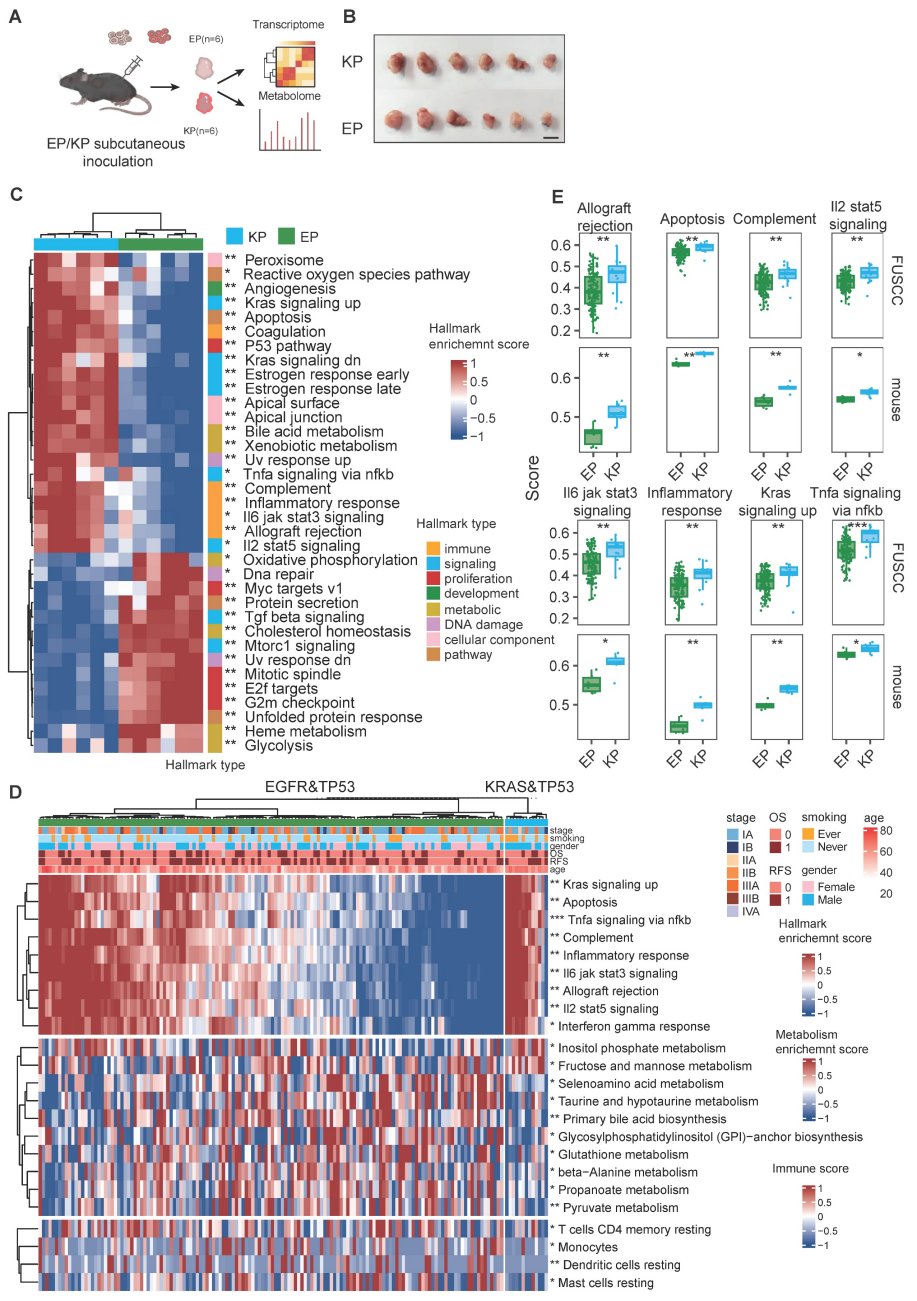
** EP cell lines mimic Oncogenic signaling and Tumor microenvironment of EGFR-mutant LUAD.** (**A**) Schematic illustration of allograft formation. EP1 cells (n = 6) and KP (n = 6) cells were subcutaneously transplanted in lower flanks of immunocompetent mice. Tumors were then collected for bulk RNA-seq and untargeted metabolome after 2 weeks. (**B**) End point illustration of EP1 and KP allograft tumors. Scale bar = 1cm. (**C**) Heatmap illustrating hallmarks with significant differences between EP and KP groups. (**D**)The heatmap presents the distribution of hallmarks, metabolic pathways, and immune cell scores that show significant differences between the EGFR&TP53 and KRAS&TP53 in FUSCC cohort. (**E**) Bar plot showing the distribution of hallmark enrichment scores for both the LC-1000 cohort and mouse subcutaneous tumors, highlighting differences between EP and KP groups. All data are mean ± SEM. *, P < 0.05; **, P < 0.01; ***, P < 0.001. Student's t-test in (**F**) and (**H**).

EGFR and KRAS represent the two most critical driver mutations in LUAD 4, 42. The establishment of the EP cell line was intended to provide a robust model that accurately recapitulates oncogenic signaling pathways and TME in EGFR-mutant LUAD. To assess this, EP1 (n = 6) and KP (n = 6) cell lines were subcutaneously implanted into immunocompetent mice. Tumors were harvested after 20 days of growth for bulk RNA-seq and metabolomic profiling (Figure 4A, B). PCA analysis revealed clear transcriptional (Figure S6C) and metabolomic (Figure S6D) separation between EP and KP tumors, while intra-group samples demonstrated clear clustering, indicating high consistency within each group. Differential gene expression analysis identified 1265/574 significantly up/down-regulated genes upon EP compared with KP (Figure S6A). To characterize the molecular features of EP, REACTOME enrichment analysis was conducted (Figure S6E), highlighting significant enrichment of EGFR-associated pathways (e.g., interactions with phospholipase C-gamma, GRB2, and SHC1 events in EGFR signaling), and reflecting the strong activation of EGFR downstream signaling in EP tumors 43. Metabolic pathways, including cholesterol biosynthesis, glycolysis, and steroid metabolism, were also significantly upregulated, underscoring a substantial difference in the metabolic program between EGFR and KRAS mutations. KRAS is a gene encoding a small GTPase 44. Oncogenic mutations of KRAS prevent the KRAS protein from hydrolyzing GTP to GDP, keeping it in an active state that continuously signals downstream pathways 45. Conversely, KP tumors were significantly enriched for GTPase-related signaling pathways, such as G alpha (q), G alpha (i), GPCR downstream signaling, and signaling by GPCR. These findings align with the biology of KRAS mutations. Notably, immune-related pathways, including immunoregulatory interactions, neutrophil degranulation, innate immune system activation, and chemokine receptor-ligand interactions, were also enriched in KP tumors, suggesting a more active immune microenvironment in KRAS-mutant LUAD compared to the immunosuppressive TME observed in EGFR-mutant LUAD.

To further explore, we analyzed transcriptomic data from EGFR; TP53 co-mutant (n = 142) and KRAS; TP53 co-mutant (n = 13) invasive adenocarcinoma patients in the FUSCC cohort (Figure 4D). We performed parallel enrichment analyses using both the mouse transcriptomic data (Figure 4C) and human data (Figure 4D) from the FUSCC cohort to demonstrate whether the molecular characteristics were consistent. In the FUSCC cohort, inter-patient heterogeneity led to considerable variation within each group. In contrast, the mouse tumors exhibited excellent intra-group homogeneity. Notably, the differential tumor-associated signaling pathways (including apoptosis, KRAS signaling, and TNFα signaling) and immune signaling pathways (such as allograft rejection, complement, IL2-STAT5 axis, IL3-JAK-STAT3 axis, and inflammatory response) observed in the mouse cohort were highly consistent with those found in the human cohort (Figure 4E).

Metabolic reprogramming is crucial for promoting tumorigenesis and shaping the immune microenvironment. Transcriptomic data reveal substantial differences in metabolic pathways between EGFR- and KRAS-mutant tumors. Untargeted metabolomics identified differential metabolites, with 224 and 459 metabolites upregulated in EP and KP tumors, respectively (Figure S6B). Functional annotation of these metabolites showed significant upregulation of beta-alanine and glutathione metabolism pathways in EP tumors, whereas amino sugar and nucleotide sugar metabolism and arachidonic acid metabolism were enriched in KP tumors (Figure S6F). Similarly, in the FUSCC cohort, ssGSEA pathway scoring for metabolic pathways indicated a marked increase in glutathione and beta-alanine metabolism in the EP group, with a higher score for amino sugar and nucleotide sugar metabolism in KP tumors, though the latter did not reach statistical significance (Figure S6G).

### Compromised adaptive immunity and M2 macrophage polarization in EGFR-mutant LUAD

**Figure 5:**
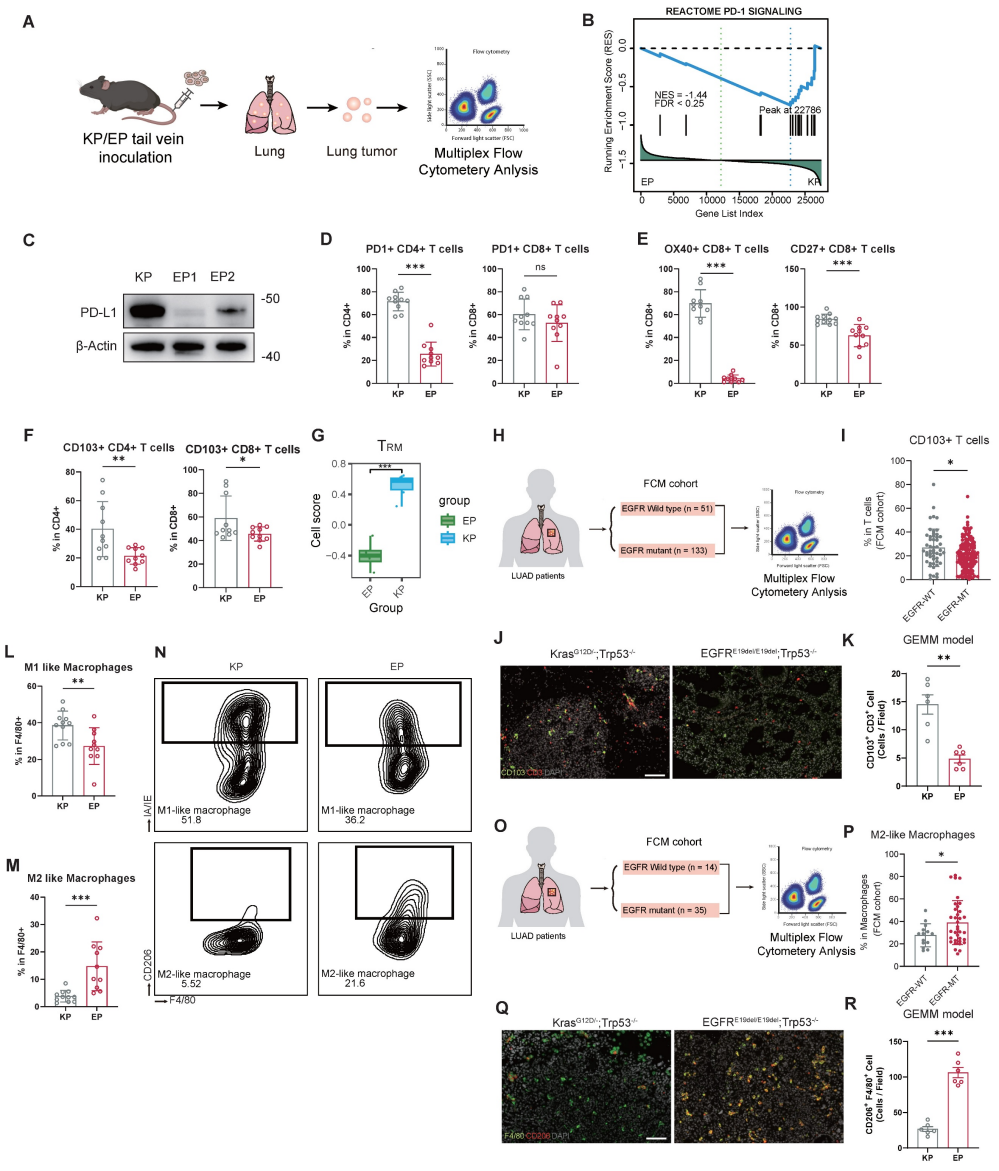
** EP model exhibits weakened adaptive immunity and M2 macrophage polarization.** (**A**) Schematic illustration of orthotopic LUAD formation. EP1 cells and KP cells were intravenously injected into the immunocompetent mice. The tumor nodules were then excised for multiplex flow cytometry. (**B**) Enrichment plot of PD-1 signaling pathway gene set. (**C**) Western blot assay of PD-L1 in KP, EP1 and EP2 cells. Loading control: β-Actin. (**D**) Bar graphs comparing the expression of PD1^+^ populations of CD4^+^ or CD8^+^ cells between KP and EP group. (**E**) Bar graphs comparing the expression of OX40^+^ (co-stimulator marker) and CD27^+^ (co-stimulator marker) populations of CD8^+^ T cells between KP and EP group (KP, n = 10; EP, n = 10). (**F**) Bar graphs comparing the expression of CD103^+^ populations of CD4^+^ or CD8^+^ cells between KP and EP group (KP, n = 10; EP, n = 10). (**G**) Bar plot illustrating the distribution of gene expression-based cell scores for T_RM_ cells in the EP and KP allografts. (**H-I**) Schematic illustration (**H**) and bar graphs comparisons (**I**) of CD103^+^ T cells populations of T cells between EGFR-WT and -MT in FUSCC Flow Cytometry (FUSCC FCM) cohort. (**J**) Representative image of IF staining for EGFR^E19del/E19del^; Trp53^-/-^ mice and Kras^G12D^; Trp53^-/-^ GEMM mice lung tumors showed infiltration of CD103^+^ (Green) and CD3^+^ (Red) cells. DAPI: Grey. Scale bar = 100μm. (**K**) Bar graph comparisons of infiltration of CD103^+^ /CD3^+^ cells in EGFR^E19del/E19del^; Trp53^-/-^ and Kras^G12D^; Trp53^-/^ group. (**L-M**) Bar graphs comparing the expression of M1-like macrophages (**L**) and M2-like macrophages (**M**) populations of macrophages between KP and EP group (KP, n = 10; EP, n = 10). (**N**) Representative gating image of M1-like macrophages (MHC-II^+^) and M2-like macrophages (CD206^+^) populations of macrophages (CD11B^+^/GR1^-^/F4/80^+^) in KP and EP group. (**O-P**) Schematic illustration (**O**) and bar graphs comparisons (**P**) of M2-like macrophages populations between EGFR-WT and -WT in FUSCC FCM cohort. (**Q**) Representative image of IF staining for EGFR^E19del/E19del^; Trp53^-/-^ mice and Kras^G12D^; Trp53^-/-^ GEMM mice lung tumors showed infiltration of F4/80^+^ (Green) and CD206^+^ (Red) cells. DAPI: Grey. Scale bar = 100μm. (**R**) Bar graph comparisons of infiltration of CD206^+^ /F4/80^+^ cells in EGFR^E19del/E19del^; Trp53^-/-^ and Kras^G12D^; Trp53^-/^ group. All data are mean ± SD. *, P < 0.05; **, P < 0.01; ***, P < 0.001. Student's t-test in (**D**), (**E**), (**F**), (**G**), (**I**), (**K**), (**L**), (**M**), (**K**), (**P**) and (**R**).

Transcriptomic data from both allografts and patient cohorts (TCGA and FUSCC) revealed substantial differences in TIME between EGFR- and KRAS-mutant LUAD. To investigate these distinctions further, we constructed orthotopic LUAD models established by tail vein injection of EP or KP cells, and performed multiplex flow cytometry analysis to evaluate the infiltration abundance and function of immune cells (Figure 5A). The prevalence of CD45^+^, T cells (CD3^+^), B cells (CD19^+^), NK cells (NKP46^+^), neutrophils (GR1^+^), or macrophages (F4/80^+^) population in the orthotopic LUAD microenvironment did not significantly differ between EP and KP groups (Figure S8A).

We observed a significant downregulation of the PD-1 signaling in the EP tumors (Figure 5B). To validate this finding, we examined PD-L1 protein in EP1, EP2, and KP cells. Remarkably, PD-L1 expression was nearly absent in both EP1 and EP2 cells (Figure 5C). Immune profiling further revealed that infiltration of PD1^+^CD4^+^ T cells significantly decreased in EP group (Figure 5D).

Despite comparable overall T cell abundance, EP tumors exhibited impaired adaptive immune activation. We examined key co-stimulatory factors (OX40 and CD27) within T cells, which are tumor necrosis factor receptor molecules. The infiltration of OX40^+^CD8^+^ and CD27^+^CD8^+^ T cells significantly decreased in EP group relative to KP group, indicating impaired T cell activation (Figure 5E). Additionally, the proportion of T_RM_ cells, CD103^+^CD4^+^ and CD103^+^CD8^+^, was significantly lower in EP group (Figure 5F). Subsequently, we performed GSVA on the transcriptomic data from the EP and KP allograft models using a signature for T_RM_ cells 46 (Figure 5G). Consistently, the GSVA scores for T_RM_ cells in the EP subcutaneous tumor were significantly lower. Analysis of the FUSCC FCM cohort further revealed reduced CD103+ T cell infiltration in tumors from EGFR-mutant LUAD patients (n=133) relative to EGFR wild-type cases (n=51) (Figure 5H and I). Furthermore, we performed IF staining on lung tumor samples from EGFR^E19del/E19del^; Trp53^-/-^ mice and Kras^G12D^; Trp53^-/-^ GEMM mice. Tumors from EGFR-mutant GEMMs showed significantly lower infiltration of CD103⁺CD3⁺ T_RM_ cells compared with Kras-driven tumors (Figure 5J and K).

Furthermore, phenotypic analysis of macrophages revealed a preferential enrichment of M2-like (CD206+) macrophages in EP group, in contrast to the significant accumulation of M1-like (IA/IE+) macrophages observed in KP group. (Figure 5L-N). CIBERSORT analysis of the transcriptomic data from allograft models confirmed a higher M2 macrophage infiltration score in EP tumors (Figure S8B). Similarly, in the FUSCC FCM cohort, EGFR-mutant LUAD (n = 35) displayed significantly more M2-like macrophages compared to EGFR wild-type tumors (n = 14) (Figure 5M and N). Also, IF demonstrated that CD206⁺ macrophages were markedly enriched in EGFR-mutant GEMMs, indicating a greater shift toward an M2-polarized macrophage phenotype (Figure 5O and P). Additional M1 macrophage markers, CD80 and CD86, were also examined. Both CD80⁺ and CD86⁺ macrophages were significantly more abundant in KP tumors compared to EP tumors, supporting the enhanced M2 polarization under the EGFR background (Figure S8C-E). Interestingly, glutamine metabolism was significantly enriched in EGFR-mutant LUAD in both mouse models and the FUSCC cohort. Correlation analysis further revealed that glutamine metabolism scores were negatively associated with infiltration of CD8⁺ T cells, while positively correlated with infiltration of M2 macrophage as estimated by CIBERSORT (Figure S8F). These findings suggest that glutamine metabolism may play a key role in shaping the immunosuppressive tumor microenvironment in EGFR-mutant LUAD.

Collectively, these findings demonstrate that the EGFR-driven LUAD exhibits an immunosuppressive tumor microenvironment, characterized by impaired adaptive immune activation and enhanced M2 macrophage polarization, which faithfully reflects the immune landscape observed in both EGFR-mutant LUAD patients and GEMM models.

## Discussion

**Figure 6:**
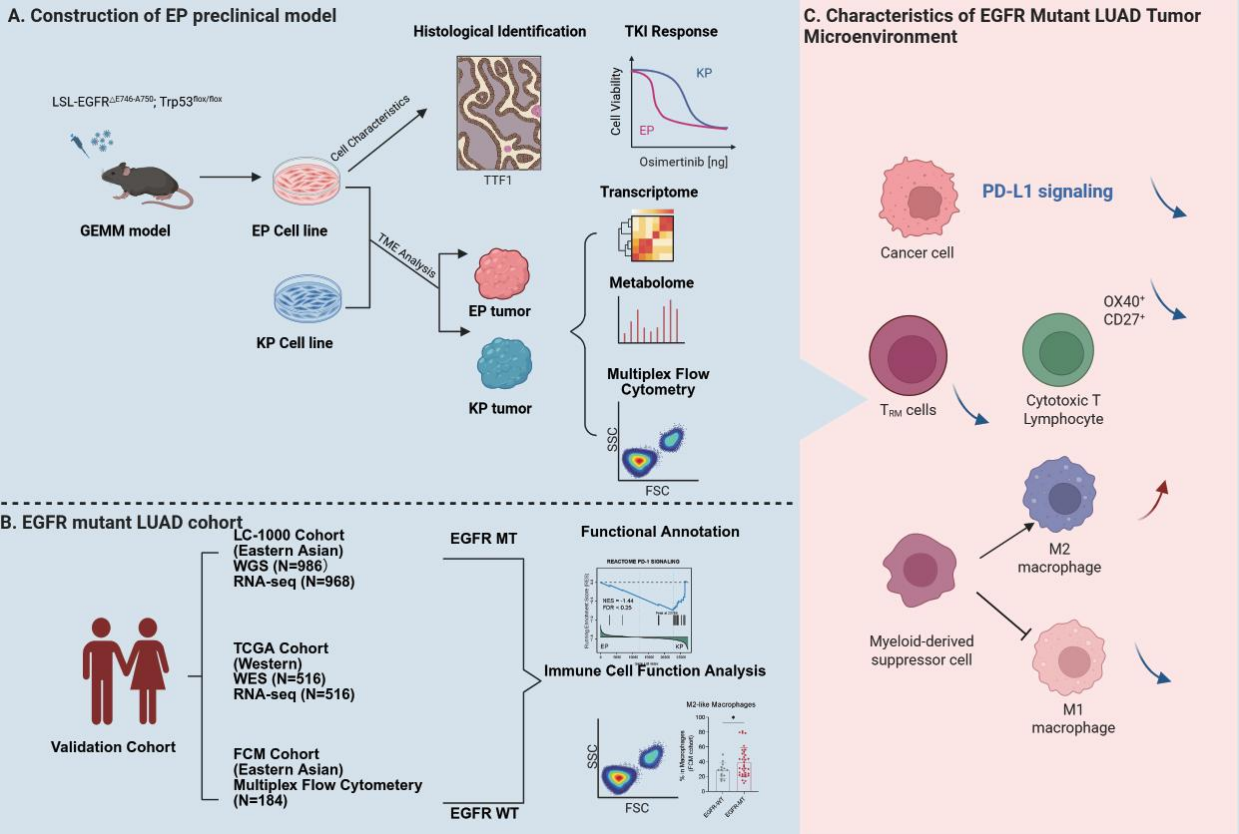
** Schematic diagram illustrating the construction of the EP model and TME characteristics of EGFR-mutant LUAD**.

There are limited preclinical models available to mimic immune dynamics and tumor microenvironment of EGFR-mutant LUAD. Herein, we constructed the GEMM-derived EP cell line, a practical model which harbors a defined *hEGFR* exon 19 deletion mutation and is capable of forming orthotopic LUAD in immunocompetent mice. Using the EP-derived orthotopic lung cancer model, we performed immune profiling in combination with multi-omics data from patient cohorts to elucidate the tumor microenvironment characteristics of EGFR-mutant LUAD (Figure 6). The observations of EGFR dependence, TKI sensitivity, and known resistance mechanisms were included primarily to demonstrate the biological fidelity and clinical relevance of the EP model.

Previous preclinical studies on immune remodeling mechanisms in EGFR-mutant LUAD have mostly leveraged models that exogenously express EGFR mutants in EGFR wild-type murine tumor cell lines, primarily (such as) LLC and MC38 47-50. For instance, Sugiyama et al. observed that EGFR-TKI treatment could improve the suppressive immune microenvironment and enhance the efficacy of PD-1 antibodies in an LLC cell line model expressing EGFR mutants 50. Similarly, Nishii et al. reported that in an MC38 model with exogenous EGFR mutation expression, combined treatment with EGFR-TKI, PD-1, and VEGFR inhibitors significantly promoted CD8^+^ T cell responses 49. However, these findings have not been substantiated by real-world data from multiple clinical trials 51-53. Both LLC and MC38 cell lines have complex genetic backgrounds, and their proliferation is not dependent on pathway activation through exogenous EGFR mutations. Therefore, utilizing these models to investigate immune remodeling mechanisms driven by EGFR mutations may introduce potential experimental artifacts and may not be an optimal choice.

The EP cell line is derived from the Cre-induced LSL-EGFR^E19del/E19del^; Trp53^flox/flox^ GEMM, which spontaneously develops LUAD. As such, it is entirely dependent on EGFR downstream signaling pathways for proliferation and growth. Furthermore, multi-omics data revealed distinctive molecular features and microenvironment characteristics of EP allograft model from KP model. Notably, these features exhibit a high degree of consistency with human EGFR-mutant cohorts. Therefore, the EP cell lines can serve as a reliable model to simulate tumor microenvironment of EGFR-mutant LUAD.

EGFR-TKI is the standard first-line treatment for EGFR-sensitive mutant LUAD. However, resistance is inevitable by the virtue of prolonged administration. The parental EP cell line demonstrates strong sensitivity to Osimertinib both in vitro and in vivo. Extending Osimertinib exposure induces acquired resistance in EP cell, which can also be reproduced in EP allograft models. Consequently, the EP model is highly suitable for investigating the mechanisms of TKI resistance. Based on transcriptome and histological analysis, we found EMT, PI3K-AKT and WNT signaling pathway highly enriched in EP-res tumor. Our findings are highly consistent with clinical reports that EMT (~13-15%) and PI3K-AKT activation (~10-15%) account for a substantial subset of EGFR-TKI resistance cases 54-56. Also, its ability to form tumors in immunocompetent mice makes it an ideal model for exploring the crosstalk between TKI resistance and the immune microenvironment.

We observed an increase in M2-like TAMs, as indicated by CD206 upregulation, within EP tumors. This finding corroborates prior reports that CD206+ TAMs suppress T-cell activation by hindering antigen presentation 57, pointing to a potential pathway for local immune suppression. Additionally, T_RM_ cells—responsible for rapid responses to tumor-associated antigens—were significantly decreased in EP tumors 58. This finding was corroborated by our multiplex flow cytometry data and supported by an independent single-cell transcriptomic cohort 16. Previous studies have reported that tumor-driven upregulation of glutamine/glutathione metabolism can generate a markedly immunosuppressive tumor microenvironment, including suppression of T cell infiltration and function as well as promotion of M2 macrophage polarization 59-61. According to our transcriptome and metabolism analysis, glutathione metabolism activity is significantly upregulated in EGFR-mutant LUAD.

Multiple clinical trials have indicated that patients with EGFR-mutant LUAD show limited response to α-PD1 immunotherapy, regardless of whether EGFR-TKI is administered. Consistently, our in vivo experiments demonstrated that EP-res tumors exhibited no significant response to PD-1 blockade, either as monotherapy or in combination with Osimertinib. Gene set enrichment analysis revealed that the PD-1 signaling pathway is downregulated in the EP group tumors. Additionally, the EP cell line exhibits minimal PD-L1 expression at the protein level. In our immune profiling, we observed a lower overall enrichment of PD1^+^CD3^+^ T cells in the EP group compared to the KP group, suggesting that immune evasion in EGFR-mutant LUAD is not regulated through the PD-1/PD-L1 axis. Therefore, identifying novel mechanisms of immune evasion in EGFR-mutant LUAD is of great significance for developing new treatments for patients with EGFR-mutant LUAD. And the EP model may serve as a robust and practical platform for further investigation.

The human EGFR protein differs significantly in both sequence and structure from its murine counterpart, which can result in strong antigenicity when introduced into mice. This antigenicity is attributed to interspecies differences that can lead to immune rejection of cells expressing hEGFR mutants in immunocompetent mice. To address this, our EP orthotopic or allograft models were established in Cre-negative LSL-EGFR mice, which share the C57BL/6 background. This approach avoids immune rejection while preserving the integrity of the model for studying immune dynamics and tumor interactions.

## Supplementary Material

Supplementary figures and tables.

## Abbreviations

LUAD: lung adenocarcinoma
GEMM: genetically engineered mouse model
EMT: epithelial-mesenchymal transition
T_RM_: tissue-resident memory T
TKIs: tyrosine kinase inhibitors
mPFS: median progression-free survival
ICI: immune checkpoint inhibitor
TME: tumor microenvironment
PBS: phosphate-buffered saline
μCT: micro-computed tomography
FUSCC: Fudan University Shanghai Cancer Center
WGS: whole genome sequencing
PCA: principal component analysis
DEGs: differentially expressed genes
TIME: tumor immune microenvironment
α-PD1: anti-PD1 antibody
