# Supplementary material for: A novel mouse cell line model reveals the tumor intrinsic and immune characteristics of EGFR-mutant lung cancer
Source: Theranostics. 2026 Jan 1;16(2):1006–22. doi: 10.7150/thno.118234 (PMC12675003; doi:10.7150/thno.118234)
Supplement: Supplementary file 1 — Supplementary figures and tables. [file thnov16p1006s1.pdf]

Supplementary materials

This PDF file includes:

Supplementary Figures. S1 to S8

Supplementary Tables. S1 to S3

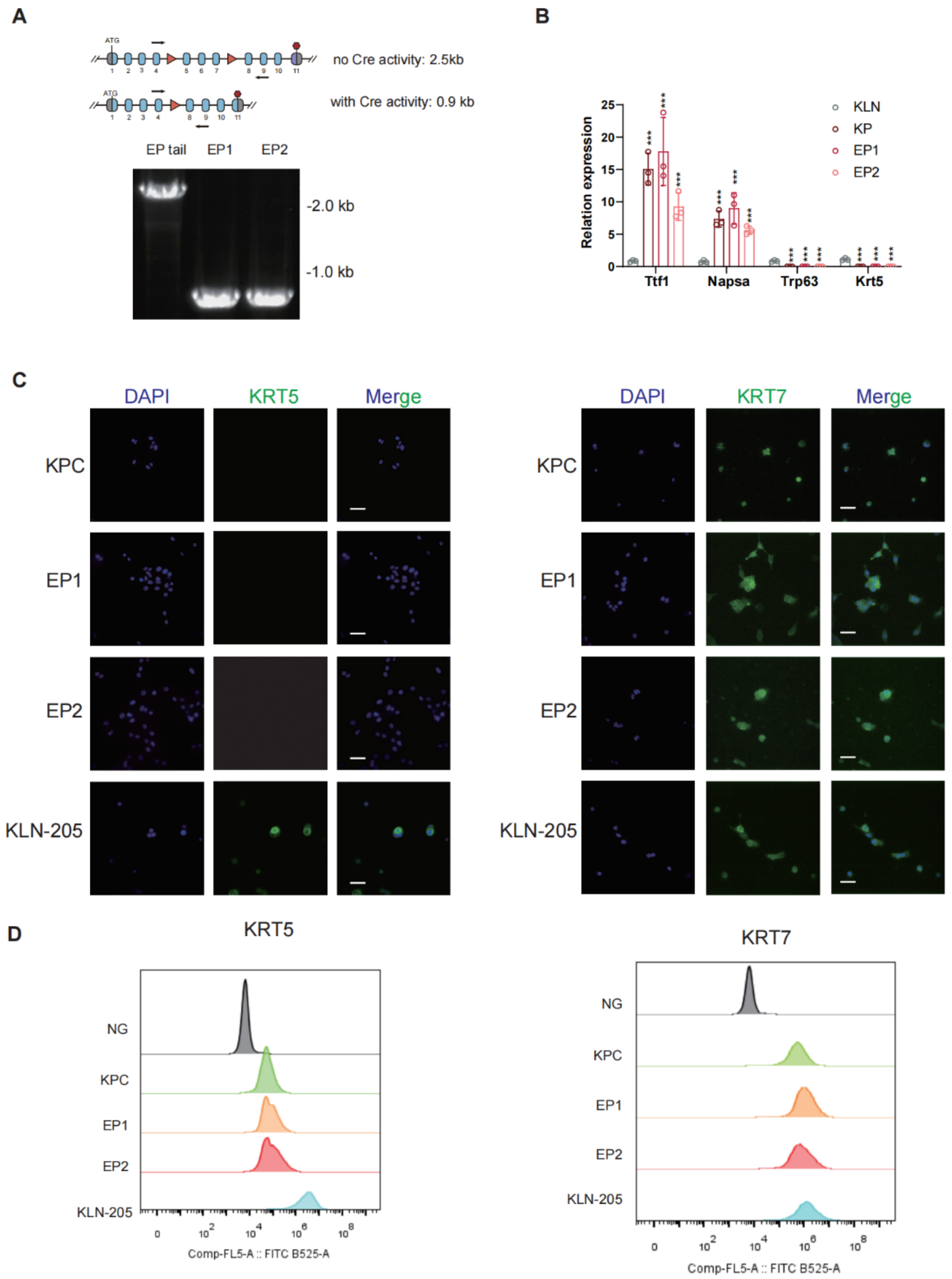

Figure S1. Characterization of EP cell lines.

(**A**) PCR analysis confirming the truncation mutation of Trp53 in EP1 and EP2 cells. The primer design was presented in the top of the panel. (**B**) qPCR analysis of adenocarcinoma

marker (Ttf1, Napsa) and squamous carcinoma marker (Krt5, Np60) in EP1, EP2, KP and KLN cell lines. (**C**) Immunofluorescence staining of KRT5 (left panel) and KRT7 (right panel) in EP1, EP2, KP, and KLN205 cells. n = 3. Scale bar = 25 μm. (**D**) Flow cytometry analysis of KRT5(left panel) and KRT7 (right panel) expression in EP1, EP2, KP, and KLN205 cells.

All data are mean ± SD. *, $P < 0.05$; **, $P < 0.01$; ***, $P < 0.001$. One-way ANOVA with Dunnett's multiple comparison test in (**B**).

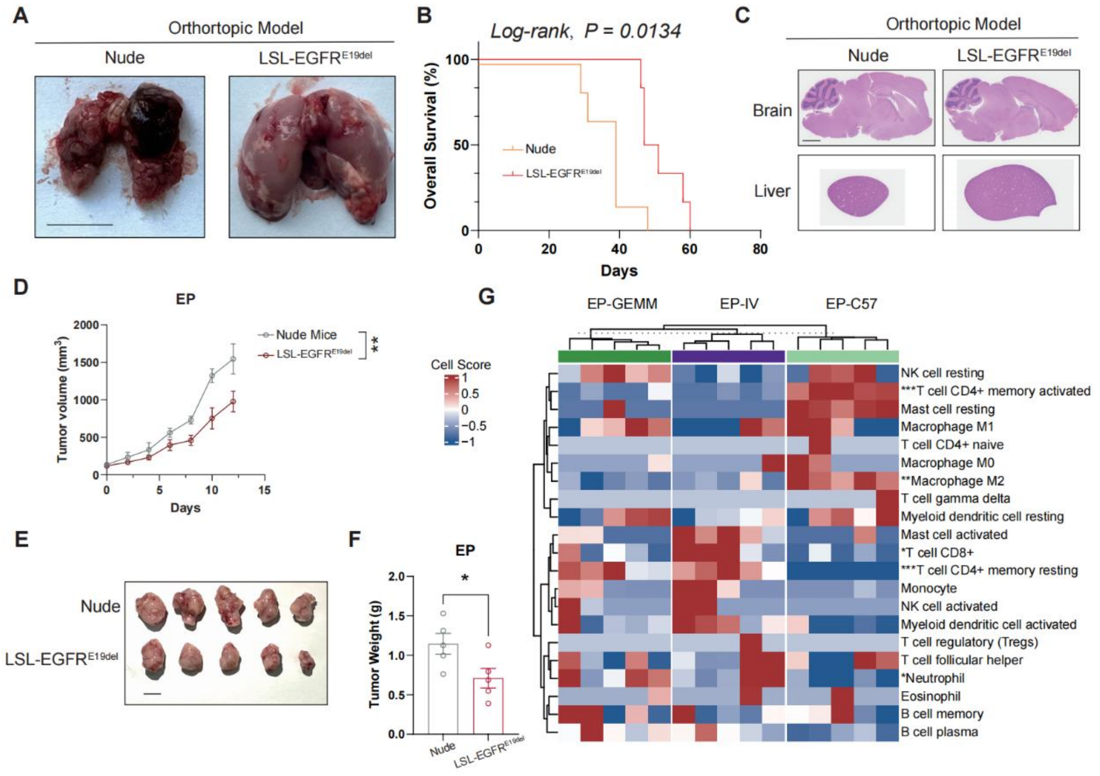

Figure S2. Comparison of EP1 tumorigenesis in immunocompetent versus immunodeficient mice.

(**A**) Representative endpoint images of lung tumors formed by tail vein injection of EP1 cells into immunocompetent and immunodeficient mice. Scale bar = 1 cm. (**B**) Kaplan–Meier survival analysis of immunocompetent (n = 6) and immunodeficient (n = 6) mice following tail vein injection of EP1 cells. (**C**) Representative H&E staining of liver and brain sections from immunocompetent and immunodeficient mice injected with EP1 cells via the tail vein. Scale bar = 1000 μm. (**D–F**) Tumor growth curves (**D**), endpoint tumor images (**E**), and tumor weights (**F**) of subcutaneous implantation of EP1 cells in immunocompetent and immunodeficient mice. (**G**) The immune microenvironment composition of the EP1 model

was analyzed based on RNA-seq. Heatmaps illustrate the microenvironmental characteristics of subcutaneous tumor models, tail vein models in immunocompetent mice, and spontaneous tumor models in GEMM model.

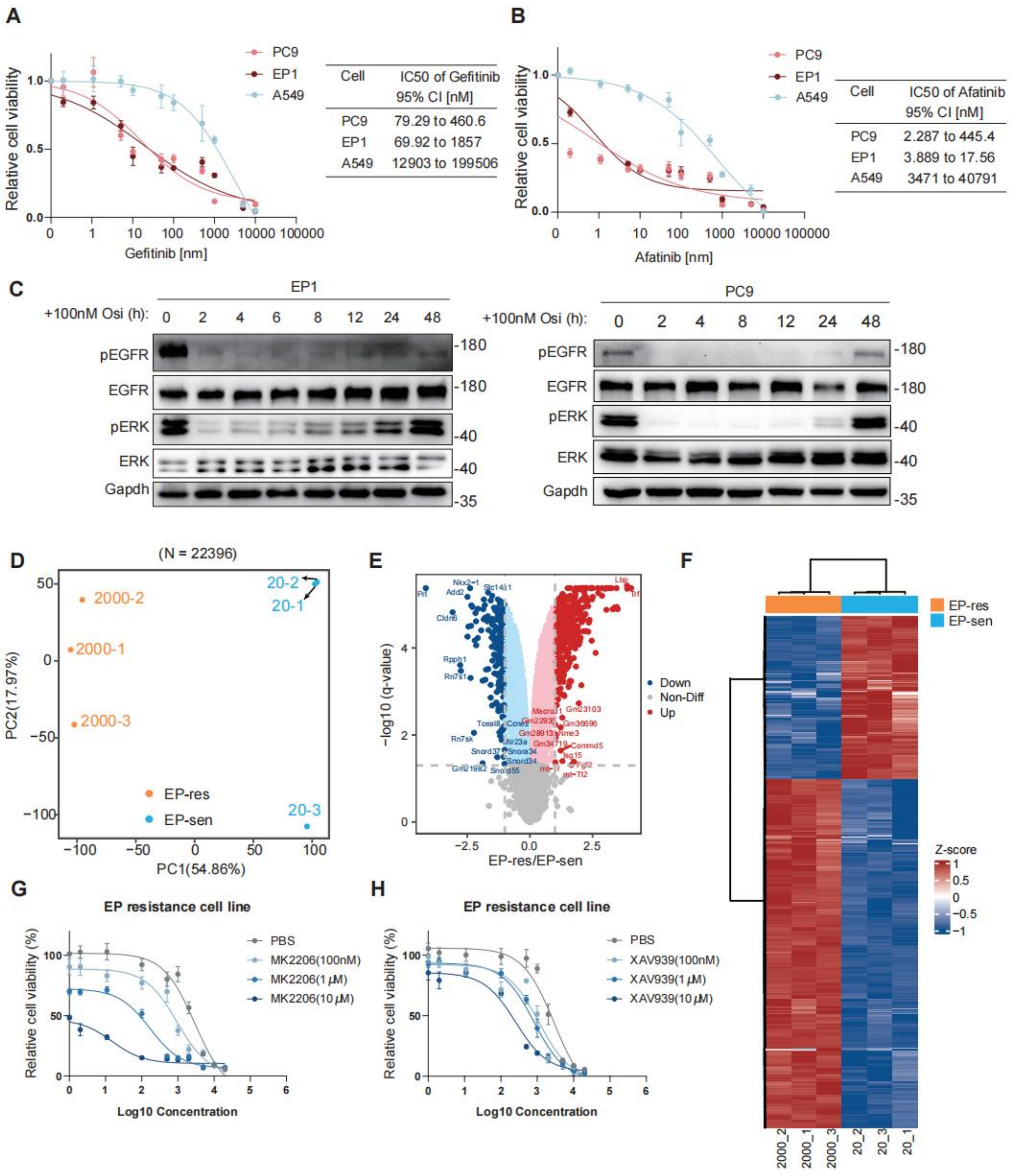

Figure S3. EP cell lines simulate the response of EGFR-TKI in EGFR-mutant LUAD in vitro.

(**A**) Dose response curve of PC9, EP1 and A549 treated with Gefitinib. The IC50 value with

95% confidence interval of Osimertinib was presented in the right of the panel. n = 2. (**B**) Dose response curve of PC9, EP1 and A549 treated with Afatinib. The IC50 value with 95% confidence interval of Osimertinib was presented in the right of the panel. n = 2. (**C**) Osimertinib treatment assays for inhibiting EGFR signaling pathway in EP1 (left) and PC9 (right) cells. Cells were treated with Osimertinib (100nM) and the protein expression of phosphorated and total EGFR and ERK at indicated time intervals was analyzed by Western blot. Loading control: β-actin. (**D**) PCA clustering results for Ois-resistance (EP-res) and sensitive (EP-sen) samples based on gene expression. (**E**) The volcano plot illustrates the distribution of DEGs between the Osi-resistant and sensitive groups, with the horizontal axis showing the log2 FC and the vertical axis displaying the -log10(q-value). Genes upregulated in the resistant group are marked in red, while those upregulated in the sensitive group are shown in blue. (**F**) Heatmap displaying the expression profiles of DEGs between Osi-resistant and sensitive EP cell line groups. (**G**) Dose-response curves were generated for EP-res cells co-treated with Osimertinib and the PI3K-AKT pathway inhibitor MK-2206, the latter applied across a range of concentrations (0, 100 nM, 1 μM, 10 μM). (**H**) Dose-response curves were generated for EP-res cells co-treated with Osimertinib and the Wnt pathway inhibitor, XAV939, the latter applied across a range of concentrations (0, 100 nM, 1 μM, 10 μM).

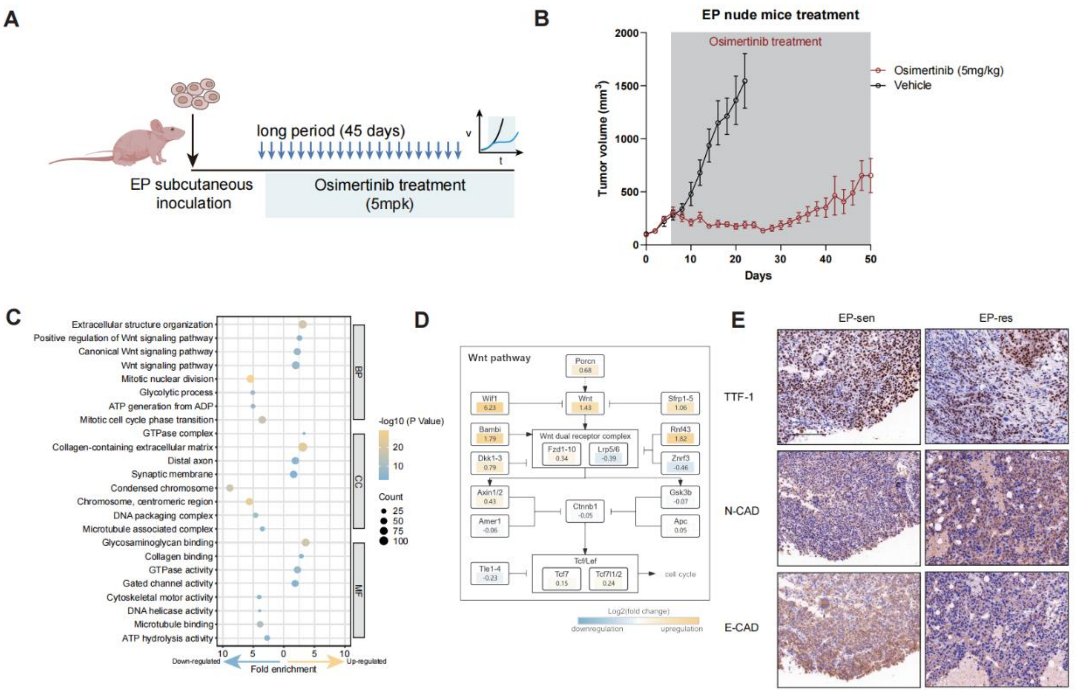

Figure S4. Construction and characterization of Osimertinib-resistant EP tumors.

(**A-B**) Schematic illustration (**A**) and growth curve (**B**) of EP1 allograft treated with prolonged Osimertinib (Osi) administration. EP1 cells were subcutaneously transplanted in lower flanks of nude mice. Mice were subsequently treated with vehicle or Osimertinib (Osi, 5 mpk, every two days) for 45 days. (**C**) Functional enrichment results of DEGs between the resistant and sensitive groups. (**D**) Fold changes in gene expression of the Wnt pathway in the resistant group compared to the sensitive group. (**E**) Histopathological images of serial subcutaneous tumor sections injected with EP-sen and EP-res cells, showing IHC analysis for TTF-1, N-Cadherin (NCAD) and E-Cadherin (ECAD). Scale bar = 100 µm.

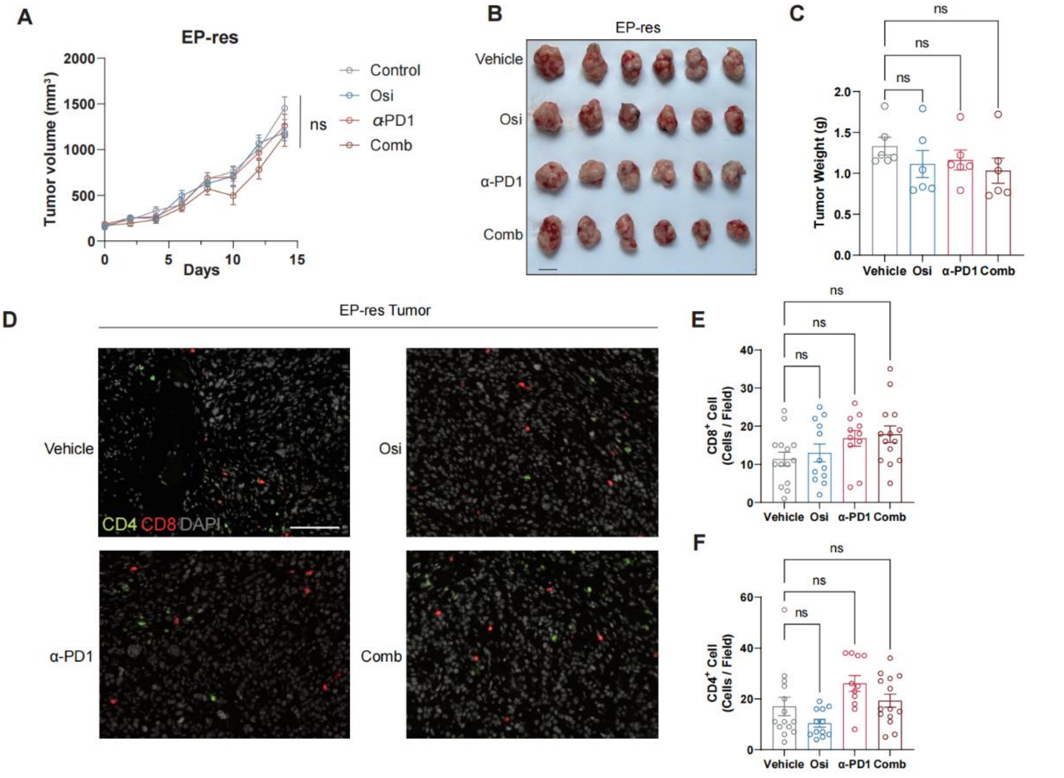

Figure S5. Limited efficacy of Osimertinib and PD-1 blockade in EP-resistant tumors

(**A-C**) Growth curve (**A**), end point illustration (**B**), and tumor weight (**C**) of EP-res allograft treated with Osi, anti-PD-1 antibody (α-PD1), or their combination (Comb). (**D**) Representative image of IF staining for EP-res tumors treated with vehicle, Osi, α-PD1 or Comb showed infiltration of $CD4^+$ (Green) and $CD8^+$ (Red) cells. DAPI: Grey. Scale bar = 100μm. (**E-F**) Bar graph comparisons of infiltration of $CD8^+$ cells (**E**) and $CD4^+$ cells (**F**) in vehicle, Osi, α-PD1 and Comb group. All data are mean ± SEM. ns, not significant. Two-way ANOVA with Tukey's test in (**A**). One-way ANOVA with Dunnett's multiple comparison test in (**C**), (**E**) and (**F**).

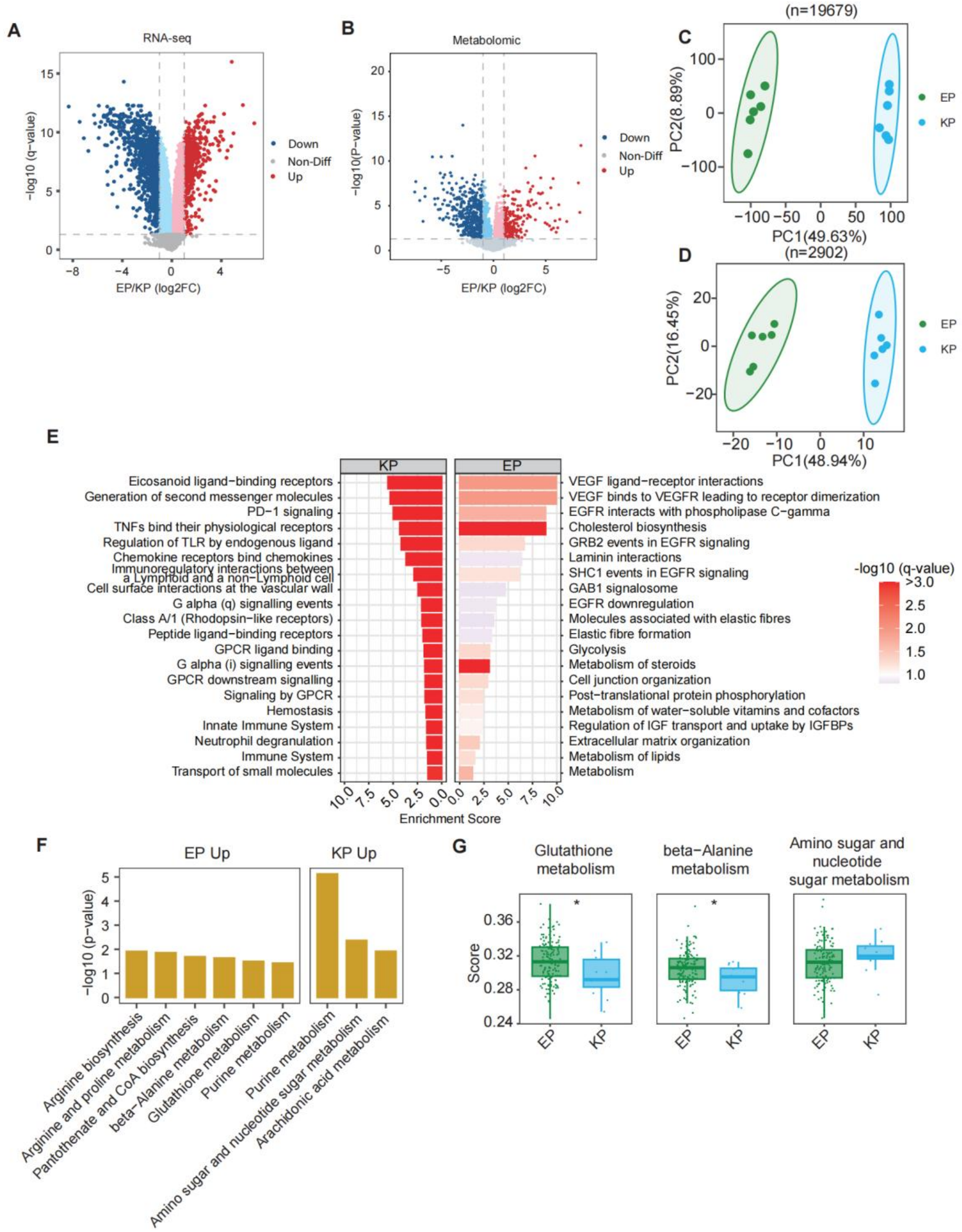

Figure S6. Reactome and biological function characteristics of the EP and KP groups.

(**A**) The volcano plot displays the distribution of DEGs between the anti-EP and KP groups, with the horizontal axis showing the log2 FC and the vertical axis displaying the -log10(q-value). Genes upregulated in EP group are highlighted in red, while those upregulated in KP

group are shown in blue. (**B**) The volcano plot displays the distribution of differentially metabolites between the anti-EP and KP groups, with log2 (FC) on the horizontal axis and -log10 (p-value) on the vertical axis. Metabolites upregulated in the EP group are highlighted in red, while those upregulated in the KP group are shown in blue. (**C**) PCA clustering results for EP and KP samples based on gene expression and (**D**) metabolite abundance. (**E**) The bar plot illustrates the Reactome pathways enriched for upregulated genes in both the EP and KP groups, with the horizontal axis representing the enrichment score and colors indicating the FDR value. (**F**) Metabolite enrichment pathways that are upregulated in EP and KP groups of mouse subcutaneous tumors. (**G**) Bar plot displaying the metabolic pathways in gene EP and KP groups from the FUSCC cohort, based on enrichment results derived from (**F**).

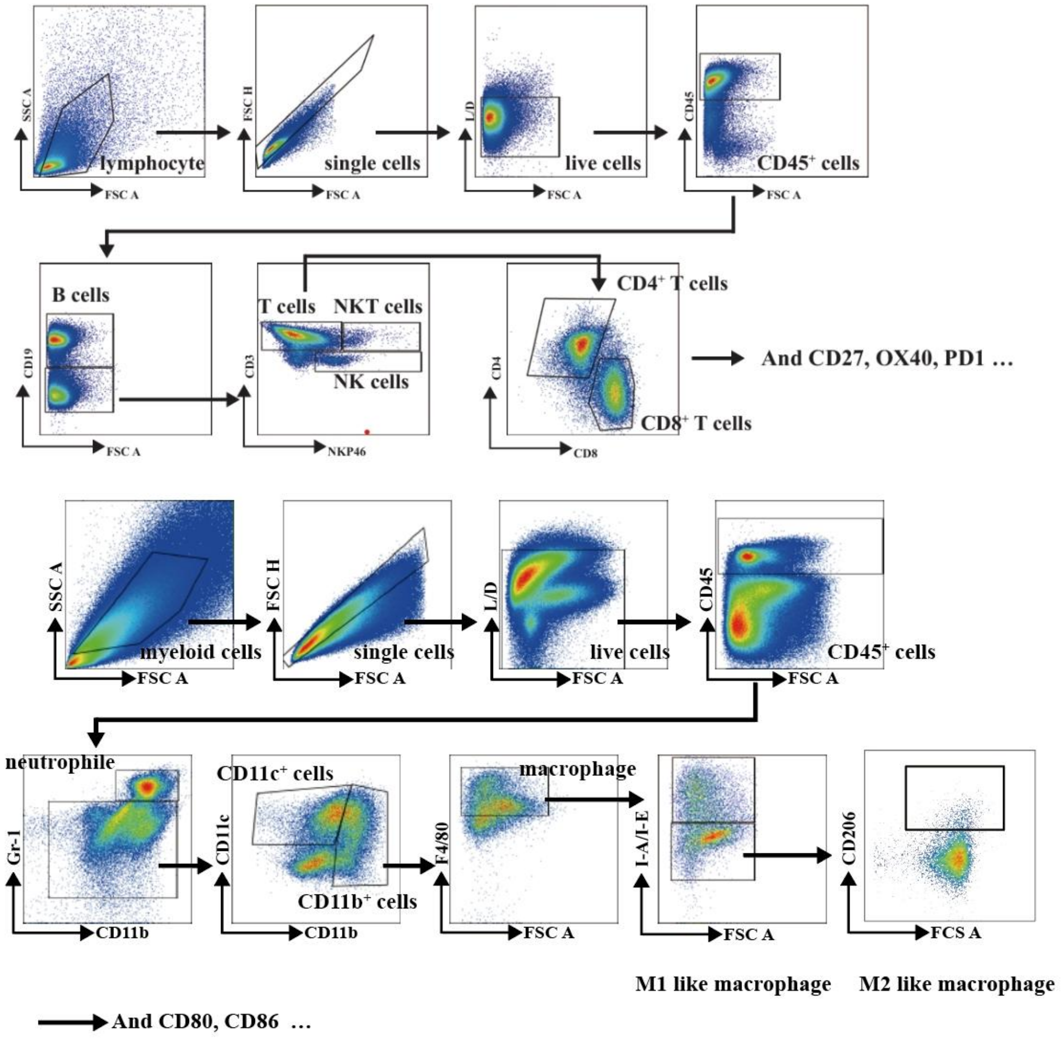

Figure S7. Representative gating strategy applied for immune cell profiling in this study.

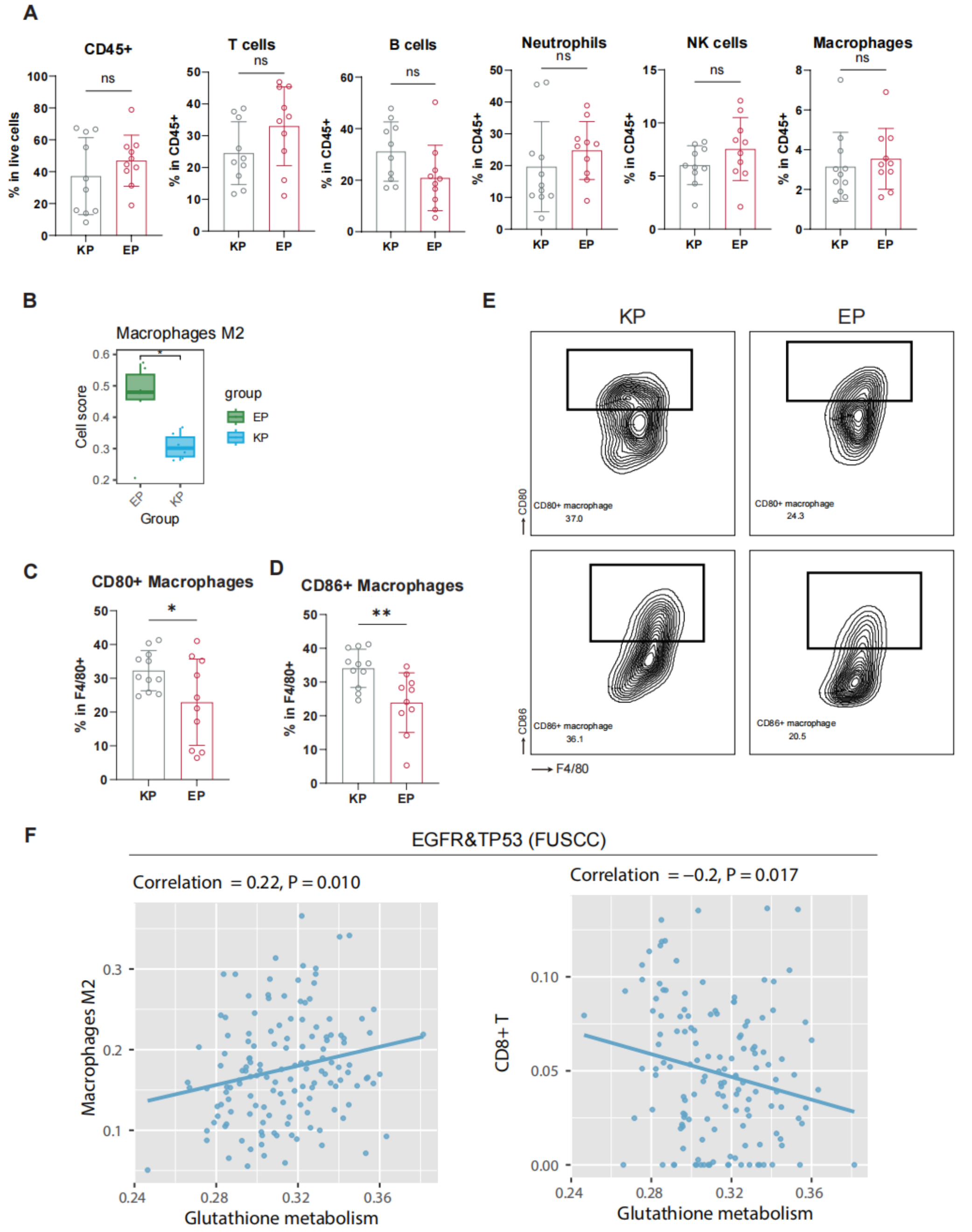

Figure S8. EP Model Exhibits Weakened Adaptive Immunity and M2 Macrophage Polarization, related to Figure 5.

(**A**) Bar graphs comparisons of $CD45^{+}$ populations of live cells and comparisons of T cells

($CD3^+$), B cells ($CD19^+$), neutrophils ($GR1^+$), NK cells ($NKP46^+$) and macrophages ($F4/80^+$) of $CD45^+$ cells between KP and EP group (KP, n = 10; EP, n = 10). (**B**) Bar plot depicting the distribution of M2 macrophage cell scores based on gene expression in the EP and KP groups. (**C-D**) Bar graphs comparisons of $CD80^+$ (**C**) and $CD86^+$ (**D**) populations of macrophages ($CD11B^+/GR1^-/F4/80^+$) in KP and EP group (KP, n = 10; EP, n = 10). (**E**) Representative gating image of $CD80^+$ and $CD86^+$ macrophages populations of macrophages ($CD11B^+/GR1^-/F4/80^+$) in KP and EP group. (**F**) Scatter plots separately illustrate the correlation between glutamine metabolic pathway activity and M2 macrophages as well as CD8+ T cells. All data are mean ± SD. *, $P < 0.05$; **, $P < 0.01$; ***, $P < 0.001$. Student's T test in (**A**), (**B**), (**C**) and (**D**).

Table S1. Baseline characteristics of FUSCC FCM cohort.

	**EGFR mutant** (N=133)	**EGFR wild-type** (N=51)	**P-value**
**Age**			
Mean (SD)	62.3 (9.52)	62.9 (9.53)	0.705
**Gender**			
Female	87 (65.4%)	19 (37.3%)	**<0.001**
Male	46 (34.6%)	32 (62.7%)	
**Smoking status**			
No	104 (78.2%)	28 (54.9%)	**0.0031**
Yes	29 (21.8%)	23 (45.1%)	
**Tumor size > 2cm**			
No	55 (41.4%)	16 (31.4%)	0.282
Yes	78 (58.6%)	35 (68.6%)	
**T stage**			
T1	106 (79.7%)	32 (62.7%)	0.122
T2	20 (15.0%)	14 (27.5%)	
T3	5 (3.8%)	4 (7.8%)	
T4	2 (1.5%)	1 (2.0%)	
**N stage**			
N0	99 (74.4%)	33 (64.7%)	0.065
N1	9 (6.8%)	1 (2.0%)	
N2	25 (18.8%)	17 (33.3%)	
**M stage**			
M0	132 (99.2%)	50 (98.0%)	1
M1	1 (0.8%)	1 (2.0%)	
**TNM stage**			
I	94 (70.7%)	31 (60.8%)	0.0834
II	11 (8.3%)	1 (2.0%)	
III	27 (20.3%)	18 (35.3%)	
IV	1 (0.8%)	1 (2.0%)	
**CTR**			
Mean (SD)	0.839 (0.202)	0.898 (0.206)	0.0837
**VPI**			
PL0	114 (85.7%)	39 (76.5%)	0.201
PL1-3	19 (14.3%)	12 (23.5%)	
**LVI**			
No	89 (66.9%)	32 (62.7%)	0.719
Yes	44 (33.1%)	19 (37.3%)	

CTR: Consolidation tumor ratio

VPI: Visceral pleural invasion

LVI: Lymphovascular invasion

Table S2. Primer and oligonucleotide sequences.

Primer	Primer Sequence	Note
**PCR primers**		
Trp53-cre-F	TCCCATCCACAGCCATCA	PCR for Trp53 cre activity
Trp53-cre-R	GGCAGGCACAAACACGAA	PCR for Trp53 cre activity
Krt5-F	CTCTGTCGTTACAAACAGTGTCT	qPCR
Krt5-R	CTTAGCCCGCTACCCAAACC	qPCR
Ttf1-F	ATGAAAGGGGGCACAAGCAAA	qPCR
Ttf1-R	TCCAAGCACTGAGAGGGACAT	qPCR
Napsa-F	CACAGGACCTAGTGAGGAGATC	qPCR
Napsa-R	AACCAGACTCCACCAAGGTGGA	qPCR
Trp63-F	CACCTGGACGTATTCCACCG	qPCR
Trp63-R	CATGGCACGGATAACAGCG	qPCR
β-Actin-F	GGCTGTATTCCCCTCCATCG	qPCR
β-Actin-R	CCAGTTGGTAACAATGCCATGT	qPCR
**Genotyping Primers**		
Trp53-genotype-F	GAGCATGGAAGTAAGACCCCTTCT	Genotyping primers
Trp53-genotype-R	GACAGGGTTTCTCTATGTAGCCCT	Genotyping primers
EGFR-Mut-F	GCTGATCCGGAACCCTTAAT	Genotyping primers
EGFR-Mut-R	TCCTCTGATGATCTGCAGGTTT	Genotyping primers
EGFR-WT-F	AGTCGCTCTGAGTTGTTATCAG	Genotyping primers
EGFR-WT-R	TGAGCATGTCTTTAATCTACCTCGATG	Genotyping primers

Table S3. Primary antibodies performed in multiplex flow cytometry analysis.

Product name	Target	Clone	Fluorochrome	Catalog	Vendor	RRID
Brilliant Violet 785™ anti-mouse CD45 Antibody	CD45	30-F11	BV785	103149	BioLegend	AB_2564590
FITC anti-mouse CD3ε Antibody	CD3	145-2C11	FITC	100306	BioLegend	AB_312670
PerCP/Cyanine5.5 anti-mouse CD4 Antibody	CD4	RM4-5	percp/cy5.5	100540	BioLegend	AB_893326
APC/Fire™ 750 anti-mouse CD8b.2 Antibody	CD8	53-5.8	APC/Fire750	140420	BioLegend	AB_2819885
PE/Dazzle™ 594 anti-mouse CD19 Antibody	CD19	6D5	PE-dazzle594	115554	BioLegend	AB_2564000
PE/Cyanine7 anti-mouse CD335 (NKp46) Antibody	NKP46	29A1.4	PE/cy7	137617	BioLegend	AB_11218594
PE/Cyanine7 anti-mouse CD134 (OX-40) Antibody	OX40(CD134)	OX-86	PE/cy7	119415	BioLegend	AB_2566155
Brilliant Violet 605™ anti-mouse/human CD11b Antibody	CD11B	M1/70	BV605	101257	BioLegend	AB_11126744
Brilliant Violet 650™ anti-mouse CD11c Antibody	CD11C	N418	BV650	117339	BioLegend	AB_2562414
PerCP/Cyanine5.5 anti-mouse Ly-6G/Ly-6C (Gr-1) Antibody	GR1(Ly6G/Ly6C)	RB6-8C5	percp/cy5.5	108427	BioLegend	AB_893558
APC anti-mouse/rat/human CD27 Antibody	CD27	LG.3A10	APC	124211	BioLegend	AB_2073425
Brilliant Violet 605™ anti-mouse CD279 (PD-1) Antibody	PD-1	29F.1A12	BV605	135220	BioLegend	AB_2562616
Alexa Fluor® 700 anti-mouse CD103 Antibody	CD103	2E7	AF700	121442	BioLegend	AB_2813992
PE anti-mouse F4/80 Antibody	F4/80	BM8	PE	123110	BioLegend	AB_893486
APC anti-mouse CD80 Antibody	CD80	16-10A1	APC	104713	BioLegend	AB_313135
PE/Cyanine7 anti-mouse CD86 Antibody	CD86	A17199A	PE/cy7	159207	BioLegend	AB_3106036
FITC anti-mouse I-A/I-E Antibody	IA/IE	M5/114.15.2	FITC	107605	BioLegend	AB_313320
Brilliant Violet 421™ anti-mouse CD206 (MMR) Antibody	CD206	C068C2	BV421	141717	BioLegend	AB_2562232
